# A novel cable bacteria species with a distinct morphology and genomic potential

**DOI:** 10.1128/aem.02502-24

**Published:** 2025-04-22

**Authors:** Anwar Hiralal, Philip Ley, Jesper R. van Dijk, Cheng Li, Dmitrii Pankratov, Jiji Alingapoyil Choyikutty, Galina Pankratova, Jeanine S. Geelhoed, Diana Vasquez-Cardenas, Clare E. Reimers, Filip J. R. Meysman

**Affiliations:** 1 Research Group Geobiology, Department of Biology, University of Antwerp 98888, Antwerp, Belgium; 2 Department of Functional and Evolutionary Ecology, University of Vienna, Vienna, Austria; 3 College of Earth, Ocean and Atmospheric Sciences, Oregon State University 97120, Corvallis, Oregon, USA; 4 Integrated Science and Technology, School of Integrated Sciences, James Madison University 3745, Harrisonburg, Virginia, USA; 5 Biological & Ecological Engineering, Oregon State University, Corvallis, Oregon, USA; 6 Department of Biotechnology, Delft University of Technology 2860, Delft, The Netherlands; University of Delaware, Lewes, Delaware, USA

**Keywords:** cable bacteria, microbial diversity, extracellular sheaths, periplasmic ridges, closed genome, *Ca*. Electrothrix yaqonensis

## Abstract

Cable bacteria form a group of multicellular prokaryotes that enable electron transfer over centimeter-scale distances within marine and freshwater sediments. To this end, the periplasm of these filamentous bacteria contains specialized conductive fibers, which extend along the full length of each filament and incorporate a novel Ni-containing NiBiD cofactor. Currently, the cable bacteria include two recognized genera, *Candidatus* Electrothrix and *Candidatus* Electronema, but the genetic and morphological diversity within the clade remains underexplored. Here, we report the isolation and characterization of a novel cable bacteria species from an intertidal estuarine mudflat within Yaquina Bay (Oregon, USA). A clonal enrichment culture of a single strain (designated YB6) was generated, and filaments were subjected to genomic, morphological, spectroscopic, and electrical characterization. Strain YB6 shares key physiological traits with other cable bacteria, such as long-distance electron conduction and the presence of the nickel bis(dithiolene) cofactor. At the same time, YB6 exhibits distinctive morphological features, including pronounced surface ridges that are up to three times wider than in other cable bacteria. Additionally, filaments are extensively enveloped by extracellular sheaths. Genomic analysis reveals that strain YB6 harbors metabolic pathways and genes found in both the *Ca*. Electrothrix and *Ca*. Electronema genera. Phylogenetic and phylogenomic analyses indicate that strain YB6 represents a novel species (average nucleotide identity <95%) that forms an early branch within the *Ca*. Electrothrix clade. The proposed name is *Ca*. Electrothrix yaqonensis sp. nov., honoring the Yako’n tribe of Native Americans whose ancestral lands encompassed Yaquina Bay.

**IMPORTANCE:**

This study expands our understanding of the genetic and morphological diversity of cable bacteria, a group of prokaryotes with a unique metabolism based on long-range conduction. We present the detailed morphological and genomic characterization of a novel species: *Ca*. Electrothrix yaqonensis, strain YB6, isolated from an intertidal estuarine mudflat. Importantly, the strain exhibits a distinctive ridge morphology (harboring the conductive fibers) and abundant formation of extracellular sheaths. Genomic analysis reveals that YB6 shares metabolic features with both *Ca*. Electrothrix and *Ca*. Electronema genera.

## INTRODUCTION

Cable bacteria are a group of filamentous bacteria that perform electrogenic sulfide oxidation (e-SOx) within the upper layers of aquatic sediments (1–4). Their electrogenic metabolism involves a peculiar division of labor between cells in the multicellular filament: electrons are transferred from deeper sediment layers, where cells perform sulfide oxidation, to the surface, where other cells use oxygen or nitrate as the terminal electron acceptor (2, 5). This long-distance electron transport is facilitated by a specialized network of conductive periplasmic fibers, which are embedded within parallel-running ridges that span a filament from end-to-end (6, 7). These fibers are interconnected via a conductive cartwheel-shaped structure present at the cell–cell interfaces, thus providing redundancy to the electrical network (8). Conductance within the fibers is facilitated by a nickel bis(dithiolene) (NiBiD) complex, a novel cofactor that is unique to cable bacteria and does not share any homology with other biological cofactors (9, 10). The presence of this nickel-based NiBiD cofactor is supported by genomic analysis, which reveals genetic adaptations for nickel cycling in cable bacteria (11).

Phylogenetic and phylogenomic analyses indicate that marine and freshwater cable bacteria form a monophyletic clade within the *Desulfobulbaceae* family (12–14), and to date, 25 species of cable bacteria have been described (11, 12, 14–17). These are currently classified into two major genera: *Candidatus* Electrothrix, which consists of saltwater species, and *Candidatus* Electronema, which consists of freshwater and brackish species (14, 17). However, two unclassified filamentous organisms (designated AR3 and AR4) are positioned between *Ca*. Electrothrix and *Ca*. Electronema in both phylogenomic and 16S rRNA gene trees and may represent new cable bacteria genera (12, 18). Recent 16S rRNA analysis suggests that the cable bacteria diversity has been underestimated and may encompass up to 90 different species (19).

The characterization of novel cable bacteria species is challenging because cable bacteria cannot be cultured axenically. Initially, cable bacteria were cultivated in enrichment incubations starting from natural sediment, which produced enrichments containing multiple species and/or strains (12–14, 18). Recently, a method for “clonal culturing” was developed (20), which enriches for a single strain in autoclaved sediment. Clonal culturing facilitates the achievement of closed genomes through metagenome assembly (15) and has become the new standard for characterizing novel species of cable bacteria (11, 16, 17). To date, closed genomes have been acquired in this way for six cable bacteria species (11, 15–17). Most importantly, clonal culturing allows detailed microscopic and spectroscopic characterization of filaments from a single species and thus allows to establish an unambiguous link between genetic identity and other traits.

Despite the broad global distribution of cable bacteria (3), their diversity remains underexplored in dynamic habitats, such as estuarine and brackish environments (19). In 2017, bacterial filaments with morphological features resembling cable bacteria were detected on the anode of a benthic microbial fuel cell deployed in the Yaquina Bay estuary (21). 16S rRNA analysis of the microbial community suggested that these filaments might be taxonomically distinct from known cable bacteria species (21, 22).

In this study, we describe a clonal, single-strain enrichment culture (designated strain YB6) of cable bacteria derived from sediment in Yaquina Bay, Oregon, USA. Comprehensive microscopic and spectroscopic techniques were employed to investigate its morphology and physiology. Additionally, a closed genome of strain YB6 was generated using a combination of long-read and short-read sequencing. These genomic data were utilized to assess the strain’s metabolic potential and to conduct phylogenomic and phylogenetic comparisons to other known cable bacterium species. Our findings reveal that strain YB6 represents a novel cable bacterium species with distinctive morphological features. Its genome also provides new insights into the boundaries between established cable bacteria genera.

## MATERIALS AND METHODS

### Sediment sampling and characterization

The sediment used for incubation was collected from two mudflats in Yaquina Bay, Oregon, USA (Idaho Flat, 44.618344°N, −124.041247°W, Sally’s Bend, 44.625278°N, −124.044722°W) located close to a site where cable bacteria were detected previously (22, 23). These sediments consisted mostly of fine sand containing 25%−35% silt and clay (24). Both sampling sites are within the intertidal zone of the estuary and experience seasonal variations in salinity due to changes in freshwater inflow (27‰−30‰ from November to April and 30‰−32‰ from May to October) (25, 26). Sediments from 0 to 20−50 cm depths were collected using a hand shovel in the summer of 2019 (15°C air temperature) at low tide. Sediments were transported to Oregon State University and stored in sealed plastic buckets at 5°C with a layer of overlying water. Before being used in incubations, the sediment was sieved through a 0.5 mm mesh size metal screen to remove macrofauna and shell debris.

To determine porosity, glass petri dishes of a known volume were filled with wet sediment, weighed, and dried at 100°C for 72 h. The porosity was calculated from the weight loss of water and the total dish volume, accounting for the salt content of the pore water. Dried samples were then finely ground with a mortar and pestle. The solid-phase density was determined by adding a known mass of dried sediment to a graduated cylinder and measuring the resulting volume change. Additionally, subsamples were processed to evaluate the total carbon (TC) and total nitrogen (TN) using an elemental analyzer (Vario MACRO cube; Elementar, Germany) (27). To determine inorganic carbon (IC), ground subsamples were first reacted with concentrated phosphoric acid (85%) for 12 h to evolve CO_2_. Evolved CO_2_ was extracted from septum-sealed sample vials using a double-needle gas headspace sampler (Finnigan GasBench-II) with a continuous stream of helium gas and analyzed using an isotope ratio mass spectrometer (DELTA V, Thermo Scientific) (28). The organic carbon (OC) content was calculated by subtracting the IC from the TC. All TC, OC, IC, and TN values are reported as weight percentage and corrected for the salt content of the dried sediment. All sediment characteristics were measured in triplicate using subsamples from the two collection sites.

### Generation of a clonal enrichment culture

The clonal enrichment culture was developed following a similar procedure as previously described (15, 20). First, an enrichment culture was made with natural sediment, thus increasing the natural population of cable bacteria toward higher densities. The initial enrichment culture was prepared with sediment from Sally’s Bend, whereby sieved sediment was packed into 100 mL graduated media storage bottles (VWR) and incubated in glass fish tanks filled with aerated artificial seawater (Instant Ocean) at a salinity of ~30 at room temperature. Subsequently, a clonal culture was generated by inoculating autoclaved sediment with a limited number of cleaned filaments from the enrichment culture as described by Li et al. (29). For the clonal culturing, sieved sediment from Idaho Flat was autoclaved for 1.5 h at 121°C under an N_2_ atmosphere and stored at room temperature for 1–2 weeks before usage. Upon usage, the stored autoclaved sediment was gently mixed and transferred into 100 mL glass containers. Serial transfers of cable bacteria bundles were performed to the newly autoclaved sediment every 2 months over the course of 14 months (thus increasing the chance of achieving clonality). Polycarbonate core liners (10 cm high and 3.6 cm inner diameter, closed at the bottom with a rubber stopper) were used instead of glass containers for subsequent transfers. To determine the number of cable bacteria strains and hence verify clonality, we applied 16S rRNA V3-V4 amplicon sequencing. The final (13th generation) clonal enrichment (designated strain YB6) was used for microscopic, electrical, and spectrochemical analyses, as well as metagenome sequencing.

### Cable bacteria activity via microsensor depth profiling

Cable bacteria activity can be determined from depth profiles of geochemical indicators (O_2_, H_2_S, and pH) and electric potential (EP) in sediment incubations (2, 30, 31). The distinct geochemical fingerprint of e-SOx was confirmed by microsensor depth profiling (pH, H_2_S, O_2_, and EP) following the methods outlined previously. Microsensor calibration was carried out as described by Malkin et al. (4). The pH data are reported on the total pH scale, and ΣH_2_S was calculated from H_2_S based on the pH values measured at the same depth using the R package AquaEnv (32). The EP signal was measured using a reference electrode following the method outlined by Damgaard et al. (33), and current density was calculated as:


$$J=\varphi_{sed}(\Delta EP/\text{d}x),$$

with *J* being the current density (mA/m^2^), *φ*_sed_ being the conductivity of the bulk sediment, and ∆EP/dx the slope of the EP depth profile at the oxic–anoxic interface, where the current density is expected to be maximum. The conductivity of the bulk sediment is calculated using the Marelac R package (34) by taking the porosity and tortuosity of the sediment into account.

### Filament isolation

Individual cable bacteria filaments were picked from clonal enrichments by hand using custom-made glass hooks (29) for microscopy imaging, Raman spectroscopy, conductivity measurements, and full-length 16S rRNA gene sequencing. To remove residual sediment particles, picked filaments were washed in MilliQ (MQ) water, except before transmission electron microscopy (TEM), when filaments were cleaned in artificial seawater.

### Microscopic imaging

Bright field microscopy was performed on filaments from the clonal enrichment culture (strain YB6) using a Zeiss Axioplan 2 epifluorescence microscope with a Cool LED pE-300 light source. Images were taken with a Qimaging EXi Blue camera, and image processing was performed using Image-Pro Insight (Media Cybernetics, USA) and ImageJ software (35).

Fluorescence *in situ* hybridization (FISH) was performed on filaments of strain YB6, with Cy5-labeled probe DSB706 targeting members of the *Desulfobulbaceae* (36–38) following a FISH procedure for cable bacteria as described previously (39). Briefly, picked filaments were transferred to a polycarbonate filter (0.22 µm; Merck Isopore, Germany), fixed with a 50% ethanol solution for 5 min, and washed with MQ. Afterward, filaments were hybridized and counterstained with 2 µg/mL 4′,6- diamidino-2-phenylindole (DAPI).

To determine the presence of the distinctive ridges observed on the outer surface of cable bacteria (2, 7), scanning electron microscopy (SEM) was performed. To this end, filaments were placed in a drop of MQ onto a 13 mm polycarbonate membrane filter which was mounted on an SEM aluminum stub (1 cm diameter) with a carbon adhesive pad. After air-drying, a thin layer of gold was evenly sputtered (Polaron E5100 sputter coater) for 30 s at 13 mA and 0.13 mbar. The samples were then examined using a Phenom ProX SEM operated at 10 keV (Phenom-World B.V).

To obtain additional topological information, filaments were examined with atomic force microscopy (AFM). To this end, picked filaments were transferred to a droplet (~2 µL) of MQ on a piece of gold-coated silicon wafer (50 nm gold layer, Platypus Technologies) and left to air-dry. Silicon wafers were then glued to magnetic metal disks of 20 mm diameter and 1 mm height using double-sided carbon stickers. Imaging was performed on an XE-100 atomic force microscope (Park Systems) in tapping mode, using an aluminum SPM probe with a tip radius of less than 10 nm (AppNano ACTA-200) and with a nominal spring constant of 13–77 N/m. Topography and amplitude images were recorded and processed with Gwyddion software (40).

To obtain accurate cell width and ridge dimensions, filaments were resin-embedded, and cross sections were examined using TEM. Filaments were fixed in 2.5% glutaraldehyde in sodium cacodylate buffer (0.1 M Na-cacodylate, 3.4 mM CaCl_2_, and pH 7.4) for 30 min. The samples were then embedded in a thin layer of 2% low-melting point agarose (Fisher Bioreagents). After solidification, the agarose-embedded specimens underwent secondary fixation in 2.5% glutaraldehyde in sodium cacodylate buffer at 4°C, followed by rinsing three times for 10 min each in sodium cacodylate buffer containing 7.5% sucrose. Post-fixation was performed in 1% osmium tetroxide in 0.33 M veronal acetate with 4% sucrose (pH 7.4) at room temperature for 2 h. Subsequently, the samples were thoroughly rinsed with 0.05 M veronal acetate containing 6% sucrose (pH 7.4) and subjected to a graded ethanol dehydration series (50%, 70%, 90%, and 95% for 15 min each, followed by two changes of 100% ethanol for 40 min each). Dehydration was completed with a 30 min incubation in propylene oxide. The samples were then infiltrated overnight with a mixture of propylene oxide and Embed-812 resin (450 mL Embed-812, 450 mL dodecenylsuccinic anhydride, 450 mL N-methylacetamide, and 50 mL DMP-30; Electron Microscopy Sciences) at room temperature without an accelerator. This was followed by two 2 h incubations with pure resin without an accelerator at 37°C and a final 1 h incubation with pure resin containing an accelerator at 37°C. Polymerization was performed at 65°C for 36 h. The resulting resin blocks were sectioned using an ultramicrotome equipped with a diamond knife to yield 50 nm-thick sections. These sections were transferred to TEM grids (Electron Microscopy Sciences), stained with 3% lead citrate for 1 min, and air-dried before examination by TEM. Thin sections were imaged using a FEI Tecnai G2 Spirit BioTWIN operating at 120 kV.

### Measurement of cell and ridge dimensions

The number of ridges, the cell perimeter and the cell diameter were determined from TEM cross-section images (taken perpendicular to filament direction) using ImageJ (35). Mean values and standard deviations are reported for *n* = 10 cells. The width and thickness of four extracellular sheaths were determined from TEM cross-section images in the same way. Finally, the cell length was determined in ImageJ from light microscopy images from 10 filaments in their a native state (i.e., isolated in artificial seawater instead of MQ, and not dried). The mean and standard deviation of the cell length of *n* = 63 cells are reported. The ridge compartment width, defined as the distance between two consecutive ridge crests, was determined by dividing the perimeter of a cable bacterium cross section by the number of ridges counted (7). The cell dimensions of other cable bacterium species were also determined in the same way using cross-section images obtained by TEM or focused ion beam-SEM as reported in the literature (2, 7, 16, 41) .

### Raman microscopy

Raman spectra of cleaned filaments were acquired using a Renishaw inVia Qontor confocal Raman microscope with a 50 mW 532 nm excitation laser as described previously (9). Gratings of 1,800 L/mm were used to ensure an optimal signal-to-noise ratio response and spectral resolution of approximately 1 cm^−1^. The average laser excitation power was 5 mW, and the acquisition time was 10 s. All measurements were performed by using a ×100 objective lens (NA = 0.9) within a spectral range of 100–1,860 cm^−1^ and a thermoelectrically cooled Renishaw Centrus charge-coupled device detector at −70°C.

All recorded Raman spectra were processed in the WiRe software (v.5, Renishaw) for cosmic ray spike removal and baseline correction. The resulting individual spectra were averaged and background subtracted in OriginPro 2023 software (OriginLab). The reported spectrum hence represents the average of 25 individual spectra.

### Electrical and electrochemical characterization

All electrical and electrochemical measurements were carried out using a PalmSens4 potentiostat (PalmSens BV, Houten, the Netherlands), controlled by the PSTrace software. Intact cable bacteria filaments were deposited on interdigitated gold electrodes (IGEs; 250 × 2 digits, digit length 6,760 µm, bands/gaps 5 µm) purchased from Metrohm DropSens (Oviedo, Spain). Before the immobilization of cable bacteria, IGEs were electrochemically treated by cycling in 0.5 M H_2_SO_4_ (30 cycles from −0.2 to 1.7 V vs saturated calomel electrode, scan rate – 0.1 V/s), rinsed with MQ, incubated in 8 mM solution of 6-mercapto-1-hexanol for 24 h, washed with MQ, and dried. A bundle of cleaned cable bacterium filaments was deposited on the IGE and air-dried (similar procedure as in Pankratov et al. [42]). Conductance measurements were carried out under anaerobic conditions in a two-electrode conﬁguration, where two contacts of the IGE were connected as a combined working/reference and a counter electrode, respectively. Current (*I*)–voltage (*V*) curves were obtained by recording cyclic voltammograms between 0.2 and −0.2 V at a 10 mV/s scan rate. Electrochemical measurement of the oxygen reduction rate was performed in a standard electrochemical cell (40 mL of electrolyte solution) in a three-electrode configuration using a saturated calomel electrode and a glassy carbon rod as reference and counter electrodes. Cyclic voltammograms were obtained at a scan rate of 20 mV/s. The oxygen concentration of the electrolyte solution (50 mM phosphate buffer, pH 7.0) was adjusted by injecting an air-saturated buffer solution. The apparent Michaelis–Menten constant (*K*′*_m_*) was calculated by fitting the Michaelis–Menten equation to the experimental data (43):


$$j=j_{max}[O_{2}]/(K_{m}^{\prime }+[O_{2}]),$$

where *j*_max_ is the maximum bioelectrocatalytic current value and *j* is the current value at the corresponding oxygen concentration [O_2_].

### Full-length 16S rRNA gene sequencing and phylogenetic analysis

To determine the phylogenetic relationship of the clonal culture to other cable bacteria, individual filaments were retrieved from clonal enrichments to obtain a near full-length 16S rRNA gene (>1,450 bp) through Sanger sequencing after a nested PCR as described previously (19). To generate a 16S rRNA gene phylogenetic tree, selected full-length 16S sequences of cable bacteria and close relatives (Table S1) were aligned with MUSCLE (44). The tree was calculated with IQ-TREE (v.1.6.12) (45) using the ModelFinder option (46) and 1,000 ultrafast bootstrap iterations (47). The sequence of *Geobacter sulfurreducens* PCA (NR_075009) was used as an outgroup. Trees were visualized with FigTree (v.1.4.4). Additionally, 16S rRNA sequences of selected cable bacteria species (Table S2) were used to make a pairwise distance matrix with the distance matrix tool in MEGA (v.10.2.5).

### DNA extraction

A sediment core from a clonal enrichment with ongoing metabolic activity (as determined by microsensor profiling) was sliced to retain only the suboxic section (3–8 mm depth). Sediment from 3 to 8 mm depth was homogenized, flash-frozen in liquid nitrogen, and stored at −80°C for later use. For DNA extraction, sediment from 3 to 8 mm was thawed at room temperature and divided into aliquots of up to 0.4 g. These aliquots were processed using the RNeasy PowerSoil DNA Elution Kit, according to the manufacturer’s protocol with 10 min bead beating. Quantification of DNA was carried out utilizing the dsDNA High Sensitivity Assay with a Qubit (v.3.0) fluorometer, while quality assessment was conducted using an Implen N80 spectrophotometer (Implen GmbH, Germany). Following RNAse treatment (10 µg/mL, 15 min at room temperature) of the DNA and subsequent clean-up (DNA Clean & Concentrator−5; Zymo Research, The Netherlands), size analysis was performed utilizing a fragment analyzer (Agilent 5300), and absence of RNA was confirmed using the Qubit RNA HS Assay Kit. DNA was stored at −80°C awaiting further amplicon and metagenome sequencing.

### Amplicon sequencing to confirm clonality

As a verification of clonality, the strain diversity of cable bacteria in cultured sediment cores was assessed as described previously (11). Briefly, amplicon sequencing was performed on extracted DNA, targeting the V3–V4 16S rRNA region. The resulting sequencing reads were processed and merged using the DADA2 (v.1.26) package in R (48), and taxonomy was assigned to the merged reads using the SILVA (v.138.1) database (49).

### Metagenomics via Nanopore and Illumina sequencing

To characterize the clonal enrichment via metagenomics, extracted DNA was sequenced using Nanopore PromethION technology, carried out at the Neuromics support facility (Flanders Institute for Biotechnology, University of Antwerp). The DNA library was prepared using the SQK-LSK114 kit according to the manufacturer’s protocol and sequenced on an R10.4.1 flow cell for 80 consecutive hours without washing or spiking. Basecalling was performed using Guppy (v.7.1.4) (dna_r10.4_e8.2_sup). Raw reads were filtered for a minimum length of 10 kb and an average *q* score of >20 using Chopper (v.0.8.0) (50). Additionally, extracted DNA was sequenced using Illumina NovaSeq (hence generating high-quality reads to polish the assembly), performed by Eurofins Genomics (Konstanz, Germany). Raw reads were filtered and trimmed using Trimmomatic (v.0.32) with the PE flag, a sliding window of 4, an average quality of 30, and a minimum length of 60 bp (51).

### Genome assembly, polishing, and annotation

The processed Nanopore reads were used as input for Flye (v.2.8.3), with the --meta and --nano_corr parameters (52). Contigs > 2 Mbp that were flagged as circular (42 in total) by Flye were assessed to be truly circular by manual inspection of mapped reads that overlapped at the start and end of contigs in Tablet v.1.21.02.08 (53). The taxonomic affiliation of circular contigs was identified using GTDB-Tk v.2.3.2, with the genome classification option (54).

The initially applied polishing procedure, as described previously (11, 16, 17), did not result in a well-polished genome (the 16S rRNA sequence in the assembly changed in composition compared to the full-length 16S rRNA gene obtained by Sanger sequencing; see above). Therefore, the whole metagenomic assembly was used as input for one round of Nanopore polishing using Racon (v.1.3.3) (55) and, subsequently, one round of Illumina polishing using Pilon (v.1.24) using the --chunksize 1000000 option (56). Automatic genome annotation was performed using the National Center for Biotechnology Information (NCBI) Prokaryotic Genome Annotation Pipeline (v.6.7) and the KofamKOALA pipeline (https://www.genome.jp/tools/kofamkoala/).

### Genome relatedness and phylogenomic analysis

To determine the delineation of the obtained YB6 genome, publicly available cable bacteria genomes (53 in total) and related *Desulfobulbales* genomes (19 in total) were used for comparison (Table S3). Both nucleotide and protein sequence files (if available) were downloaded from the NCBI genome database (accessed 1 March 2024). For some cable bacteria genomes, no protein sequence file was available (see Table S3), and hence these were annotated using Prokka (v.1.14.6) (57). Completeness and contamination metrics (Table S3) were calculated using CheckM2 (v.1.0.2) (58). Average nucleotide identity (ANI) was calculated using pyANI (v.0.3.0) with the -m fastani setting enabled (59). Protein sequences were used to calculate average amino-acid identity (AAI) and percentage of conserved proteins (POCP), using CompareM (v.0.1.2) (https://github.com/dparks1134/CompareM). For genus demarcation, the commonly used values of 65% AAI and 50% POCP were used (60, 61). ANI, AAI, and POCP matrices were visualized with scripts provided in Sereika et al. (17). To construct the maximum-likelihood phylogenomic tree, only genomes of at least medium quality (>50% completeness and <10% contamination) were used (Table S3) to increase robustness of the tree. Concatenated conserved protein sequences for phylogenomic analysis were identified and aligned using GTDB-Tk (v.2.3.2) with the “identify” and “align” options (54). A maximum-likelihood tree was calculated using IQ-TREE (v.2.2.6) (45) using 1,000 standard non-parametric bootstraps and the “ModelFinder” option to determine the best-fit model (46). The genome of *Desulfurivibrio alkaliphilus* AHT 2 was used as an outgroup. Trees were visualized with FigTree (v.1.4.4) (http://tree.bio.ed.ac.uk/software/figtree).

### Annotation and analysis of metabolic genes

Previously identified protein sequences in cable bacteria involved in sulfur, phosphate, carbon, nitrogen, nickel, and electrogenic metabolism (11, 13, 15), as well as genes involved in marine adaptation (17), were used as input sequences for identification in cable bacteria genomes, using blastp with stringent parameters for hits (>80% alignment and <1e^−40^ *E* value). Phylogenetic analysis was performed using the protein sequences of the *nhaA*, *pstA*, and *cytB561* genes, as these exhibit highly divergent sequence identities between the *Ca*. Electrothrix and *Ca*. Electronema genera. To this end, blastp similarity searches were performed against the RefSeq database of NCBI (accessed 1 September 2024), and results were filtered for hits with >80% alignment and <1e^−20^ *E* value. Alignments were made using ClustalO (v.1.2.4) with the following parameters: --max-guidetree-iterations = 100, --max-hmm-iterations = 100, --output-order = tree-order (62). Maximum-likelihood trees were calculated with IQ-TREE (v.2.2.6) (47) using 1,000 ultrafast bootstraps (47) and the ModelFinder option (46). Trees were visualized with FigTree (v.1.4.4) (http://tree.bio.ed.ac.uk/software/figtree).

## RESULTS

### Development of a clonal culture of strain YB6

Sediment from two estuary sites of (near) marine salinity in Yaquina Bay (Sally’s Bend and Idaho Flat) was used for culturing: initial enrichment of natural cable bacteria was performed in sediment from Sally’s Bend, while subsequent clonal culturing was performed with sediment from Idaho Flat. Both sediments showed comparable solid-phase densities of 2.6–2.7 g/cm^3^, porosities of 0.5–0.6, organic carbon contents of 0.51%−0.69%, and molar OC:TN ratios of 7.7–8.6 (Table 1).

**TABLE 1:** Characteristics of Yaquina Bay sediment. Mean and standard deviation are reported for triplicate samples

Location	Solid-phase density (g/cm^3^)	Porosity (−)	TC (%)	TN (%)	IC (%)	OC:TN (mol/mol)
Idaho Flat	2.61 ± 0.02	0.53 ± 0.01	0.57 ± 0.01	0.066 ± 0.003	0.06 ± 0.01	7.7
Sally’s Bend	2.69 ± 0.05	0.61 ± 0.01	0.75 ± 0.04	0.080 ± 0.007	0.06 ± 0.01	8.6

After 13 generations of inoculating autoclaved sediment with cable bacteria, microsensor depth profiles revealed distinct geochemical signatures of cable bacteria activity, indicating long-distance electron transport and e-SOx (Fig. S1). After 22 days of incubation, oxygen concentrations dropped to <1 µM at a depth of 1.2 mm, while pH peaked at 8.3 near the sediment surface and dropped to 6.5 at 10 mm depth. A suboxic zone developed, devoid of detectable oxygen and sulfide (Fig. S1), with sulfide appearing ([ΣH_2_S] >1 µM) at a depth of 12 mm. Additionally, an electric potential difference ΔEP=0.17 mV across the suboxic zone (current density of 13 mA/m²) indicated electron transport. Amplicon sequencing confirmed the presence of only one amplicon sequencing variant belonging to cable bacteria (classified within the *Ca*. Electrothrix genus), indicating that the culture represented a clonal, single-strain enrichment, denoted YB6 (relative abundance of 1.7%).

### Morphology

**Fig 1:**
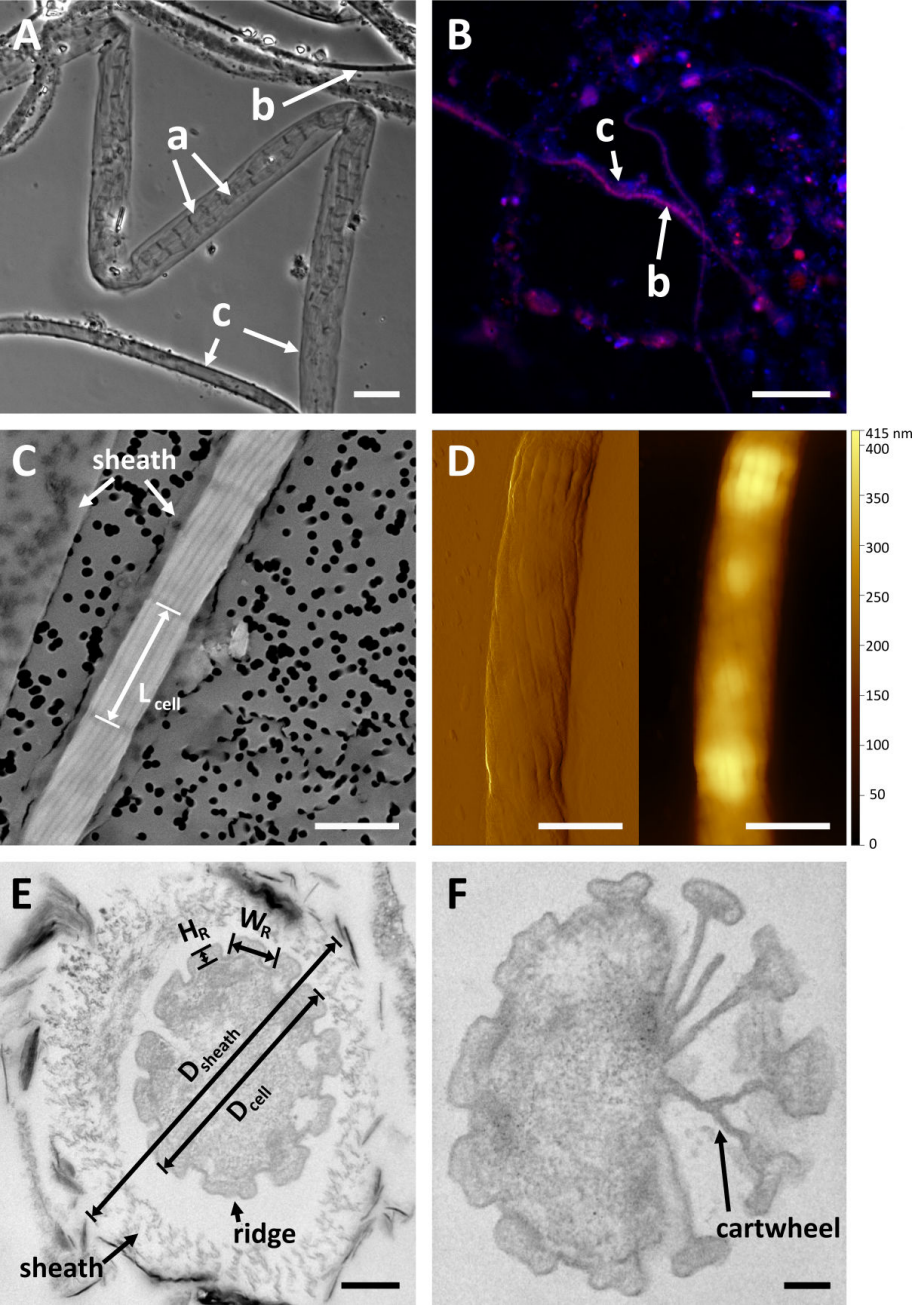
Microscopic investigation of cable bacterium strain YB6. (**A**) Light microscopy presenting presumably two partly degraded cable bacteria filaments encased within a sheath. Note that these might originate from one filament. (**B**) Cable bacteria in the overlying water assessed by FISH with the DSB706 probe (red) and counterstained with 4′,6- diamidino-2-phenylindole (DAPI, blue). Labels: a, degrading, transparent cable bacteria filaments; b, intact cable bacteria filaments; c, extracellular sheaths. (**C**) Scanning electron microscopy showcasing a YB6 cable bacterium, exhibiting pronounced ridges, enveloped in a sheath. (**D**) Amplitude (left) and topography (right) atomic force microscopy (AFM) images revealing distinct ridges traversing diagonally along the filament. The topography image of the AFM shows possible polyphosphate inclusion within the cell. (**E and F**) Transmission electron microscopy cross section of a YB6 cable bacterium surrounded by a sheath (**E**) and partial cartwheel structure revealing ultrastructural details and partial spoke (**F**), both stained with lead citrate. Scale bars: 10 µm (**A**), 20.0 µm (**B**), 1.0 µm (**C**), 1.0 µm (**D**), 0.2 µm (**E**), and 0.1 µm (**F**).

Filaments derived from the YB6 clonal culture showed the distinctive morphology of cable bacteria, characterized by ridges on the outer surface that run along the length of the filaments (Fig. 1C and D) (2, 7). FISH with probe DSB706 confirmed their taxonomic identity as members of the *Desulfobulbaceae* family (Fig. 1A). Filaments showed an individual cell length *L*_cell_ of 3.55 ± 0.63 µm (*n* = 63) and a cell diameter *D*_cell_ of 0.94 ± 0.16 µm (*n* = 10). Furthermore, TEM cross sections indicated that YB6 filaments typically possess 12 ridges (median value, *n* = 10), although some variation was noted (range: 10–13, Fig. 1E and F). These ridges are known to harbor the unique conductive fibers of cable bacteria (6, 8). At the cell–cell interfaces, the filaments display the conspicuous cartwheel structure (Fig. 1F), which is also unique to cable bacteria and is thought to provide redundancy to the electrical network (8). YB6 ridges display a rectangular shape, which is markedly different from the conical ridges seen in *Ca*. Electrothrix gigas (63). The average ridge compartment (defined as one ridge and one adjacent valley) has a width *W*_*R*_ of 228 ± 39 nm and a ridge height *H*_*R*_ of 65 ± 12 nm (*n* = 117) (Table 2; Table S4). Notably, the TEM cross-section images of YB6 filaments lack a clear unstained ridge center (Fig. 1E and F), as opposed to previously reported cross-section images of *Ca*. Electrothrix gigas (63) and *Ca*. Electrothrix communis (16). Additionally, the ridges of YB6 filaments seem to run in a spiral around the filament (Fig. 1C and D), in contrast to the parallel-running ridges typically seen in other cable bacteria filaments (6, 7).

**TABLE 2:** Comparison of the cell dimensions between YB6 and other cable bacteria species^*a*^

	YB6	*Ca*. E. gigas (63)	*Ca*. E. communis (16)	Unknown SF1 (7)	Unknown SF2 (7)	Unknown BF1 (7)	Unknown BF2 (7)	Unknown (2)	Unknown (2)	*Ca*. Electronema aureum (41)
Cell width (µm)	0.94 ± 0.16	3.1	0.6	1.1	0.6	4.0	3.9	0.8	0.8	0.9
No. of ridges	10–13	68	15	15	16	61	58	15	17	34
Ridge compartment width (nm)	228 ± 39	134	105	231	126	205	213	106	127	75

^*a*^ Values for YB6 are determined from TEM cross-sections for 10 individual cells and reported as mean ± standard deviation. Cell dimensions for other cable bacteria are taken from literature and derived from cross-sections imaged by TEM and or focused ion beam-SEM.

Conspicuously, the clonal enrichment cultures of strain YB6 consistently contained a high abundance of transparent, extracellular sheaths (Fig. 1A and B). Both empty sheaths as well as sheaths containing cable bacteria filaments were observed (Fig. 1A through C). Filaments and sheaths grew highly entangled and formed filament bundles. TEM imaging of YB6 filaments showed that the extracellular sheaths had a thickness of 219 ± 30 nm (*n* = 4) and diameter *D*_sheath_ of 1.30 ± 0.15 µm (*n* = 4), with a visible void between the cable bacteria filaments and the surrounding sheath material (Fig. 1E).

### Physiology and conductivity

**Fig 2:**
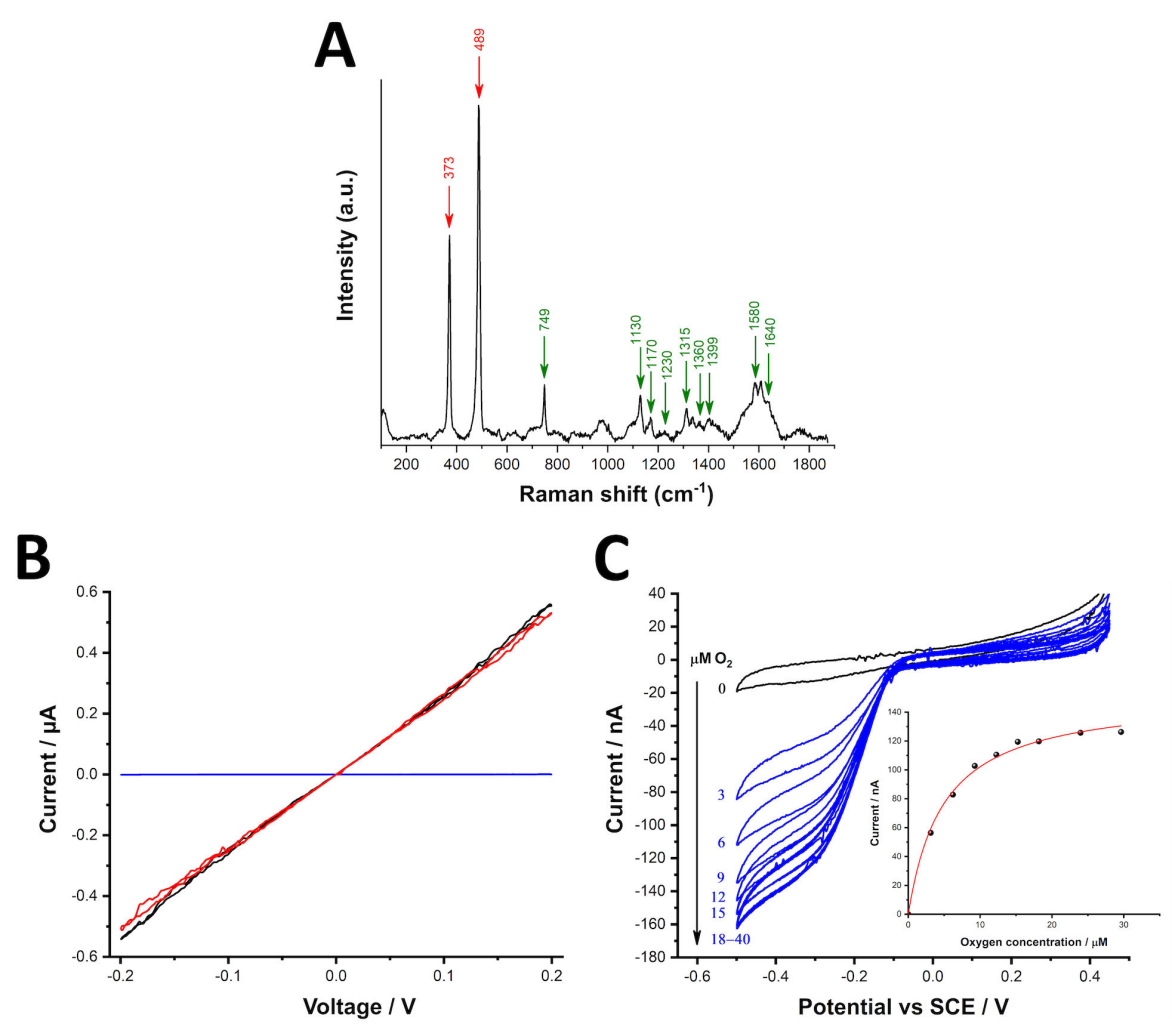
Raman spectroscopy, conductivity, and oxygen reduction measurements of cable bacterium strain YB6. (**A**) The Raman spectrum of YB6 filaments collected with a green laser (532 nm) shows a distinctive peak at 373 and 488 cm^−1^ of the NiBiD cofactor found in cable bacteria, and cytochrome signals at 750; 1,130; 1,170; 1,230; 1,315; 1,360; 1,399; 1,588; and 1,640 cm^−1^. Peak annotations can be found in Table S6. (**B**) Two consecutive *I*/*V* scans (first scan, red; second scan, black) of a bundle of YB6 filaments and one *I*/*V* scan of a bundle of extracellular sheaths (blue) deposited on an interdigitated gold electrode. (C) Representative cyclic voltammograms of YB6 cable filaments on IGE at different O_2 _concentrations of 50 mM phosphate buffer solution (pH 7.0), scan rate: 20 mV/s. Inset: current–[O_2_] plot for the presented voltammograms. SCE, saturated calomel electrode.

Cable bacteria possess a unique nickel-sulfur ligated NiBiD cofactor that produces a characteristic molecular fingerprint detectable with Raman microscopy (9, 10). Raman microscopy applied to YB6 filaments confirmed the presence of this molecular fingerprint, as revealed by scattering peaks at 371 and 488 cm^−1^ with high intensity (Fig. 2A). Additionally, the Raman spectrum revealed marked peaks at 750; 1,130; 1,170; 1,230; 1,315; 1,360; 1,399; 1,588; and 1,640 cm^−1^ (Fig. 2A), which are indicative of cytochromes, as also seen in Raman spectra of other cable bacteria species (9, 11, 15).

Conductance measurements revealed microampere currents over the −0.2 to 0.2 V range and showed that the *I*/*V* curves were linear and symmetric (Fig. 2B), thus indicating that YB6 filaments were highly conductive. In contrast, a bundle of extracellular sheaths from the YB6 culture devoid of cable bacteria filaments was not conductive (Fig. 2B). Because of the tendency of YB6 to grow highly entangled, the total filament length deposited on the IGE could not be accurately measured. Consequently, the conductance values obtained here cannot be recalculated into a conductivity estimate for individual filaments, thus preventing a direct comparison of the YB6 conductivity with other cable bacteria species (6, 42, 64). Still, the resistance values calculated from the slope of the *I*/*V* curves were ca. 380 and 360 kΩ for the first and second scans, respectively, which are comparable with earlier reported data for the bundles of extracted fiber skeletons from *Ca*. Electrothrix gigas (6, 42). This suggests that YB6 filaments show a similar conductance as other cable bacteria species. Immobilized YB6 cable bacteria bundles display a clear electrochemical activity toward oxygen reduction (Fig. 2C), similar to previously reported results for *Ca*. Electrothrix gigas (65). However, the onset potential of oxygen reduction (ca. −70 mV vs saturated calomel electrode) and the apparent Michaelis–Menten constant (*K*′*_m_*) of 4.5 ± 1.5 µM (Table S5) obtained for strain YB6 are notably different from those obtained for *Ca*. Electrothrix gigas, which are ca. 20 mV and 29.6 µM, respectively (65).

### Closed genome and evidence for a novel species

Metagenomic sequencing of the YB6 clonal enrichment culture provided 149.3 Gb of raw ONT long-read data and 7.7 Gb of raw Illumina NovaSeq PE 150 bp short-read data (Tables S7 and S8; Fig. S2). Filtering for quality and length resulted in 34.4 and 6.0 Gb for long and short reads, respectively. Assembly and polishing resulted in one circular contig representing strain YB6 classified within the *Ca*. Electrothrix genus, with a genome size of 3,738,687 bp containing two identical 16S-23S-5S rRNA loci, a guanine + cytosine (G + C) content of 51.31%, and 3,323 protein-coding sequences (Table S9). The 16S rRNA sequence was 100% identical compared to the obtained Sanger sequence.

When comparing the ANI of the acquired genome to other available cable bacteria genomes, strain YB6 is most closely related to *Ca*. Electrothrix sp. EH2 (12) with 77.82% identity and clearly represents a novel cable bacterium species using the custom threshold (<95% ANI) for species delineation (66) (Fig. S3). This is also supported by 16S rRNA sequence identity, with YB6 being most closely related to *Ca*. Electrothrix sp. EH2 with 94.95% identity (Fig. S4). This is substantially below the conventional species delineation (<98.65%) threshold but slightly above the genus delineation (>94.5%) threshold (67, 68). In addition, phylogenetic analysis of the 16S rRNA gene indicates that strain YB6 forms a separate branch between sequences of the *Ca*. Electronema and *Ca*. Electrothrix cluster, albeit with low bootstrap support (48%, Fig. S5A). Although more closely related with *Ca*. Electrothrix species, certain regions of the 16S rRNA gene of YB6 align well with sequences of *Ca*. Electronema sp. (Fig. S5B).

**Fig 3:**
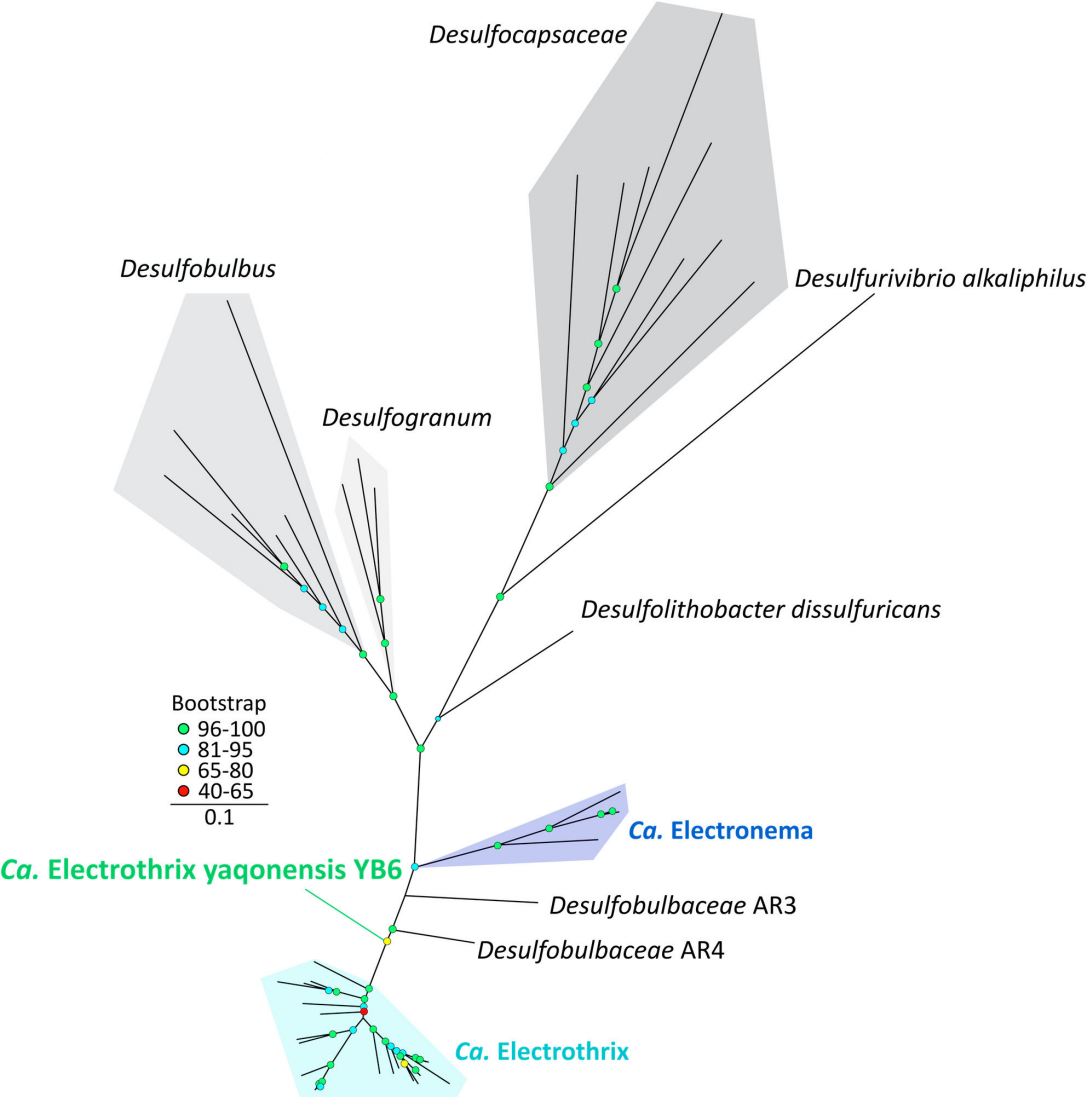
Maximum-likelihood phylogenomic tree of *Ca*. Electrothrix yaqonensis YB6 and related reference genomes. Phylogeny inferred using IQtree according to the best-fit model (LG + F + R5). *Ca*. Electrothrix yaqonensis YB6 forms a separate branch (green) from the *Ca*. Electrothrix and *Ca*. Electronema genera (shades of blue), with 68% bootstrap support (1,000 bootstraps). Only genomes of at least medium quality (>50% completeness, <10% contamination) were used as a reference.

Based on AAI, strain YB6 falls within the commonly used genus demarcation boundary of 65% (60) for both cable bacteria genera, although it displays significantly higher identities with species of the *Ca*. Electrothrix genus (74−76%) than of the *Ca*. Electronema genus (69%−70%) (Fig. S6). Similarly, a common genus demarcation value of >50% POCP (61) is reached with the *Ca*. Electronema halotolerans sp. (51%) and several *Ca*. Electrothrix spp. (50%−53%) (Fig. S6). Phylogenomic analysis with publicly available cable bacteria genomes (>50% completeness, <10% contamination; Table S3) indicates that YB6 forms a separate branch (68% bootstrap support) from the *Ca*. Electronema and the *Ca*. Electrothrix genus clusters (Fig. 3).

### Metabolic potential

**Fig 4:**
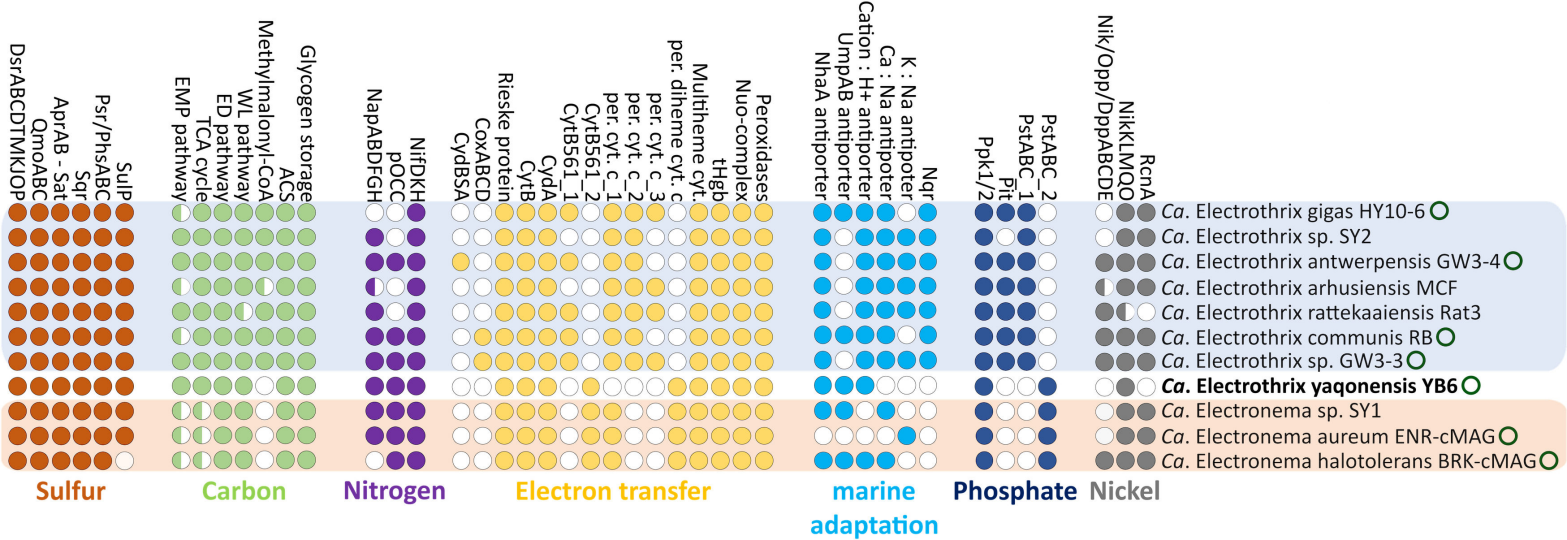
Comparison of the metabolic potential of *Ca*. Electrothrix yaqonensis YB6 and other cable bacteria. Only species representatives that have closed genomes (circle) or are of high quality (>90% completeness, <10% contamination; no circle) are shown.

The metabolic potential of strain YB6 is similar to that encoded in recently published closed cable bacteria genomes and consistent with the general metabolic model for cable bacteria that has been proposed previously (13). Yet, previously, it has also been shown that species of the *Ca*. Electrothrix genus and *Ca*. Electronema genus possess a distinct genetic repertoire in select metabolic pathways, e.g., genes involved in carbon metabolism and adaptations to the marine environment (13, 15, 17). It is therefore noteworthy that strain YB6 exhibits elements of both of these distinct genetic repertoires (Fig. 4; Table S10).

In terms of sulfur metabolism, strain YB6 codes for the reductive dsr pathway (*dsrABCDNTMKJOP*, *aprAB*, *qmoABC*, and *sat*; Table S10), as well as genes coding for a sulfide:quinone reductase and a polysulfide/thiosulfate reductase complex (Psr/PhsABC), which so far appears to be a conserved pathway in all cable bacteria (Fig. 4) (13, 15, 17). This genetic repertoire is highly similar to the sulfide oxidation pathway suggested for sulfide oxidation in *Desulfurivibrio alkaliphilus* (69).

Strain YB6 was grown with oxygen as the electron acceptor. As has been hypothesized for other cable bacteria, electrons generated from sulfide oxidation in the cytoplasm of YB6 can be transferred to the periplasm via either a Rieske-Fe-S protein, a cytochrome bc complex subunit B (CytB), and a homolog of the bd quinol oxidase subunit A (CydA) (Table S10) (13). Strain YB6 lacks a terminal cytochrome c oxidase complex (CoxACDB) or a bd quinol oxidase complex (CydBSA), which were recently discovered in some *Ca*. Electrothrix spp. and are potentially involved in oxygen reduction (15, 17). Instead, strain YB6 likely reduces oxygen through a periplasmic truncated hemoglobin (13, 65). Strain YB6 codes for one periplasmic diheme cytochrome and lacks the periplasmic monoheme cytochromes found in all other cable bacteria (Fig. 4). In addition, strain YB6 encodes for an integral diheme cytochrome (CytB561) located in the inner membrane. Phylogenetic analysis indicates that cable bacteria CytB561 sequences form two distantly related separate clusters of the *Ca*. Electrothrix and *Ca*. Electronema genera. CytB561 of strain YB6 clusters with sequences from the *Ca*. Electronema genus (Fig. S7).

The YB6 genome also contains a full *nap* operon and a putative gene encoding for a periplasmic octaheme cytochrome c (Fig. 4; Table S10), suggesting that strain YB6 can also grow with nitrate as the terminal electron acceptor through dissimilatory nitrate reduction to ammonium (DNRA) as has been shown in other cable bacteria (5, 70).

In terms of carbon metabolism, YB6 displays the potential for autotrophic growth via the Wood–Ljungdahl pathway, as seen also for other cable bacteria species (13, 65). For heterotrophic growth, strain YB6 codes for acetyl-CoA synthetase, thus putatively enabling acetate assimilation, but lacks the methylmalonyl-CoA pathway for propionate assimilation. Furthermore, strain YB6 contains the full tricarboxylic acid cycle (TCA), the Embden–Meyerhof–Parnas pathway, the Entner–Doudoroff pathway, and the reductive pentose phosphate pathway (Fig. 4; Table S10).

The YB6 genome incorporates the phosphate permease complex PstABC but lacks the sodium-dependent phosphate transporter PiT for phosphate uptake (Fig. 4). In addition, strain YB6 carries two phosphate kinase genes (ppk1 and ppk2), which indicates the potential for polyphosphate formation. Polyphosphates have been observed in other cable bacteria and could be involved in energy generation during periods of electron acceptor availability (71, 72). Phylogenetic analysis of the protein sequence of the *pstA* gene indicates two distantly related separate clusters of the *Ca*. Electrothrix and *Ca*. Electronema genera, where PstA of YB6 clusters with sequences from the *Ca*. Electronema genus (Fig. S8).

Marine cable bacteria of the *Ca*. Electrothrix genus encode the sodium quinone reductase (NQR) complex, which is widespread among marine organisms (73, 74). Interestingly, while YB6 has been isolated from a marine environment (salinity ~30), its genome lacks this NQR complex (Fig. 4). However, it does encode homologs of the Na^+^/H^+^ antiporter NhaA, homologs of the Na^+^/Li^+^/K^+^:H^+^ antiporter UmpAB, and a putative cation:proton antiporter, which were previously postulated to confer adaptations to marine environments in the brackish *Ca*. Electronema halotolerans sp. (17) (Fig. 4; Table S10). Phylogenetic analysis of the NhaA protein sequence indicates that the sequence of strain YB6 forms a cluster with those of other *Ca*. Electrothrix sequences and that the NhaA sequence of *Ca*. Electronema forms a separate branch more distantly related to *Ca*. Electrothrix sequences (Fig. S9).

Cable bacteria possess a unique nickel cofactor (9), which is accompanied by unique genetic adaptations to nickel cycling (11). The YB6 genome encodes the nickel import complex NikMKLQO, as found in all other cable bacteria, but lacks the Nik/Opp/DppABCDE transporter complex. Curiously, the YB6 genome does not possess a homolog of the nickel exporter RcnA (Fig. 4; Table S10) (75), which was previously hypothesized to transfer nickel to the periplasm in cable bacteria (11).

Finally, besides the known and common genetic repertoire, the closed YB6 genome contains 801 (out of 3,323) coding genes that are not found in any other cable bacteria genome and thus may be unique to strain YB6. Only 41 of these genes can be annotated with a known KEGG Orthology function, with the rest having no known function. This highlights the distinct, yet uncharacterizable genomic potential of strain YB6 compared to other cable bacteria.

## DISCUSSION

### YB6 exhibits a distinct morphology compared to other cable bacteria

Cable bacterium strain YB6 displays the characteristic outer ridge structures found in all known cable bacteria to date (2, 7). However, the size and shape of these ridges differ markedly from other cable bacteria (Fig. 1E and F; Table 2). The ridge compartment width (228 ± 39 nm) is considerably greater than that of other cable bacteria (Table 2), being two to three times larger than found in *Ca*. Electrothrix communis (105 nm) and *Ca*. Electronema aureum (75 nm). Only cross sections of *Ca*. Electrothrix gigas and other unidentified species showed values in the same range (Table 2) (7). Moreover, when comparing TEM cross sections, strain YB6 has a relatively low number of ridges (~12) compared to the other species (ranging from 15 to 68, Table 2). Unlike the round ridge shape seen in *Ca*. Electrothrix gigas (7, 63), *Ca*. Electrothrix communis (16), and *Ca*. Electronema aureum (41), strain YB6 shows rectangular-shaped ridge crests interspersed by defined valleys. These ridges do not reveal a visible fiber core as seen in *Ca*. Electrothrix gigas (7, 63) and sometimes rather have two or three protrusions. While this may be an artifact of the agarose or resin embedding, it is consistent with a double-fiber substructure observed in an AFM-based cell envelope investigation of cable bacteria of unknown phylogenetic origin (76).

While YB6 has a distinct ridge morphology, it clearly has the capacity for efficient long-range conduction (Fig. 2A), which is a hallmark for all cable bacteria (6). In addition, our Raman microscopy data demonstrate that YB6 also must contain the NiBiD cofactor, which is uniquely found in cable bacteria and is hypothesized to be an essential component of the conductive fiber (9, 10). Likewise, the conspicuous cartwheel-shaped structure is present in the cell–cell interfaces of strain YB6, which is thought to act as an electrical fail-safe mechanism, electrically connecting all fibers (8). Intriguingly, in one of the TEM images, one cartwheel spoke appears to end blindly without being connected to a ridge (Fig. 1D). This phenomenon has been previously observed in large cable bacteria from the Rattekaai salt marsh (7). This incomplete type of ridge development could perhaps explain the (small) variance observed in the number of ridges (10–13) across different YB6 filaments.

Another intriguing feature of strain YB6 is its ability to form extracellular, non-conductive sheaths (Fig. 1A through C, E and 2B). These tube-shaped sheaths consist of undefined nanofibers and have a ca. 50% larger diameter than YB6 filaments (Fig. 1E). Extracellular sheaths surrounding cable bacteria filaments have been previously documented in the sediments from Yaquina Bay (23) and sediments from marine Lake Grevelingen (72). An extracellular sheath could provide cable bacteria with several advantages, such as protection from predators, such as ciliates and pathogenic bacteria, or aiding motility. Investigations into the *Leptothrix* genus, which also exhibits extracellular sheath formation, reveal that sheaths are composed of interwoven nanofibrils (77). Although the sheath composition can differ between species, in *Leptothrix cholodnii*, sulfhydryl is proposed to be excreted from the cell and diffuses to the terminal ends of the filament, where the glycoconjugates form a cohesive sheath (78). However, the extracellular sheaths produced by strain YB6 and other cable bacteria require further investigation to determine their composition, biosynthesis, and functionality.

### Strain YB6 represents a novel, early branching *Ca*. Electrothrix sp.

The approach of first developing a clonal, single-strain enrichment culture followed by a combination of long-read and short-read sequencing seems crucial for obtaining closed genomes of cable bacteria species (15). Here, it has provided a closed genome of strain YB6, which is approximately 3.7 Mbp in size. Based on conventional ANI (<95%) and 16S rRNA gene sequence (<98.65%) thresholds, strain YB6 clearly represents a novel species of cable bacteria (Fig. S3 to S5). As such, it represents the seventh species with a closed genome of the 25 species with genomes acquired so far (11, 12, 15–17). The complete protologue can be found in Table S11.

Although the evidence supports that strain YB6 represents a novel species, it has been more difficult to assign strain YB6 to an established cable bacteria genus. AAI is the common measure used for genus demarcation, with a threshold set at an AAI of >65% for strains to belong to the same genus (60). Based on this criterion, strain YB6 falls in both genera, although it displays higher identities with species of the *Ca*. Electrothrix genus (74%−76%) than of the *Ca*. Electronema genus (69%−70%) (Fig. S6). Note, however, that no formal AAI demarcation boundary has been agreed upon in the microbiological community, and so some studies use lower (e.g., 63.4% in *Desulfovibrionaceae*) (79) or higher (76% in *Flavobacteriaceae*) (80, 81) demarcation values. The setting of a *Desulfobulbaceae*-specific genus boundary for AAI or, alternatively, the determination of a genus-specific ANI inflection point and alignment fraction values (82) falls outside the scope of this study but could be used in the future for more precise genus demarcation in the cable bacteria clade. Similarly to AAI, POCP is a common metric to define genus boundaries, with clusters with more than 50% POCP values belonging to the same genus (61). Again, strain YB6 displays higher than 50% POCP values with the species of both *Ca*. Electrothrix and *Ca*. Electronema genera (Fig. S6). In contrast, 16S rRNA identity indicates that strain YB6 falls within the *Ca*. Electrothrix genus (>94.5% sequence identity), but this criterion is met only for one species, and moreover, 16S rRNA has been shown not to be as robust of a criterion compared to AAI and POCP (83).

Phylogenomic analysis using concatenated conserved protein sequences from medium-quality or better *Desulfobulbaceae* genomes (>50% completeness and <10% contamination) reveals that strain YB6 forms a distinct clade, with considerable bootstrap support (>65%) for its placement (Fig. 3). The location of its branch further suggests that strain YB6 is more closely related to the *Ca.* Electrothrix clade than to the *Ca.* Electronema clade. Based on its phylogenomic position, the initial classification by GTDB-Tk (v.2) (54), and its higher ANI and AAI identity with *Ca.* Electrothrix members, we propose that strain YB6 belongs to the *Ca.* Electrothrix genus. As such, strain YB6 represents the earliest branching member of the *Ca.* Electrothrix genus, based on its basal branching position within the clade (Fig. 3).

### The metabolic potential of strain YB6 does not fit within the presumed boundaries of the *Ca*. Electrothrix and *Ca*. Electronema genera

Strain YB6 exhibits differences in its genetic repertoire for proteins involved in electron transfer compared to other *Ca*. Electrothrix species. Although strain YB6 encodes putative electron transfer complexes (Rieske-Fe-S and CytB and CydA), it lacks the periplasmic (Sec signal peptide) cytochrome c that contains a single heme-binding group and is found in all other cable bacteria (Fig. 4). Instead, strain YB6 encodes for a different periplasmic (TAT signal peptide) cytochrome c containing a diheme domain, which is otherwise only found in species of *Ca*. Electronema (Fig. 4). Cable bacteria are thought to use periplasmic cytochromes to transfer electrons in the periplasmic space to and from the conductive fibers (13). Additionally, all cable bacteria code for a putative cytochrome b561, but phylogenetic analysis indicates a clear genetic separation between the *Ca*. Electrothrix and *Ca*. Electronema copies of this gene. This genetic separation of the two clades could imply a differential ancestral origin of the protein. Strain YB6 clusters with the sequences of the *Ca*. Electronema genus (Fig. S7). Cytochrome b561 is an integral diheme cytochrome located in the cytoplasmic membrane and often involved in electron transfer systems (84), but its role in cable bacteria so far is unclear. Furthermore, our bioelectrochemical data (Table S5) suggest that strain YB6 substantially differs from *Ca*. Electrothrix gigas in terms of its onset potential for oxygen reduction (−70 mV vs 20 mV) and apparent *K*′*_m_* (4.5 µM vs 29.6 µM) (65). The link between a different genetic repertoire for cytochromes and the apparent difference in oxygen reduction between strain YB6 and other *Ca*. Electrothrix spp. requires further investigation.

A similar genetic difference is observed in the phosphate metabolism. The PstA sequence of the phosphate permease complex PstABC is phylogenetically distinct between the two genera, and PstA of strain YB6 clusters with sequences of *Ca*. Electronema and not with *Ca*. Electrothrix (Fig. S8). Moreover, the sodium-dependent phosphate transporter PiT, otherwise exclusive to the genus *Ca*. Electrothrix, is also absent in strain YB6 (Fig. 4).

While strain YB6 originates from an estuarine environment (salinity of 27–32) and was cultivated at near-marine salinity (~30), its genome does not code for an NQR complex (73), which is found in all other members of the *Ca*. Electrothrix genus (Fig. 4). It was recently hypothesized that *Ca*. Electronema halotolerans, which inhabits brackish environments (salinity 18–23), uses cation transport proteins, such as NhaA and UmpAB, which are also present in YB6, to adapt to higher salinities (17). A phylogenetic analysis of protein sequences encoded by the *nhaA* genes indicates that there are three distinct clades of cable bacteria NhaA, and YB6 NhaA belongs to a similar clade of the sequences found in the *Ca*. Electrothrix genus (Fig. S9). It is noteworthy that the NhaA sequence of *Ca*. Electronema halotolerans does not cluster with NhaA sequences of *Ca*. Electrothrix (Fig. S9), as has been previously suggested (17). Nonetheless, the lack of NQR in strain YB6 is remarkable and distinguishes the organism from other members of the *Ca*. Electrothrix genus.

Another example of the distinct metabolic potential of strain YB6 is found in its genome-encoded carbon metabolism. An earlier study has indicated that cable bacteria grow mostly autotrophically (65) by incorporation of CO_2_ through the Wood–Ljungdahl pathway (13). However, species of the *Ca*. Electrothrix genus have also shown signs of heterotrophic growth through propionate assimilation (65, 85), which can be mediated through the methylmalonyl-CoA pathway (13). The methylmalonyl-CoA pathway is present in all the high-quality genomes of the *Ca*. Electrothrix genus so far (Fig. 4), but it is lacking in the closed genome of strain YB6. Strain YB6 does encode a full Wood–Ljungdahl pathway and an acetyl-CoA synthetase. In this respect, the carbon assimilation potential of YB6 mimics that of *Ca*. Electronema sp. (17). Yet, on the other hand, the genome of YB6 includes the enzyme enolase, which is only found in some members of the *Ca*. Electrothrix genus and not in the *Ca*. Electronema genus (15, 17), and the genome also includes all genes of the TCA cycle, which is incomplete in the *Ca*. Electronema genus (17). Thus, in terms of carbon cycling, strain YB6 shows a distinct metabolic potential not found within either of the two existing genera (Fig. 4).

Strain YB6 distinguishes itself from other cable bacteria species by the absence of the nickel exporter gene *rcnA*, which facilitates the transport of nickel from the cytoplasm to the periplasm (75). In cable bacteria, the RcnA protein appears to have undergone significant modifications compared to other organisms, featuring a notably expanded histidine-rich loop, predicted to reside in the cytoplasm (11). This loop is thought to be involved in nickel binding (75) and thus could potentially enhance the transport of nickel to the periplasm. Such a role aligns fully with the presence of the NiBiD cofactor within the periplasmic conductive fibers of cable bacteria (9, 10). Interestingly, Raman microscopy of strain YB6 filaments confirms the presence of the NiBiD cofactor (Fig. 2A), making the absence of *rcnA* in YB6 somewhat surprising. However, this absence could be explained by the higher potentially bioavailability of free nickel in strain YB6’s native environment, allowing sufficient nickel to be readily accessible for NiBiD production. Detailed data of nickel concentrations in Yaquina Bay are lacking, but one study reported an average free Ni pore water concentration of ~3 µM across multiple sampling sites in Yaquina Bay (86), which exceeds the nickel levels found in other sediments inhabited by cable bacteria (87). This suggests that strain YB6 may not require the active nickel cycling machinery seen in species from nickel-poorer environments.

Overall, strain YB6 stands out from all other described cable bacteria species in terms of its metabolic potential, containing a genetic repertoire that is a mix of both the *Ca*. Electrothrix and *Ca*. Electronema genera. Despite its closer relationship to the *Ca.* Electrothrix genus, as indicated by ANI, AAI, and phylogenomic analyses, strain YB6 exhibits a metabolic profile that, in some cases, more closely resembles that of *Ca.* Electronema spp., particularly in areas such as electron transfer, phosphate uptake, carbon metabolism, and adaptation to the marine environment (Fig. 4). Given this unique metabolic potential and its intermediate phylogenomic position, strain YB6 appears to represent an early branching lineage. Its non-conformist metabolic traits highlight the complex evolutionary dynamics within the cable bacteria clade and suggest a broader functional and ecological diversity within this clade than previously recognized.

### Conclusion

In this study, we present the clonal culture YB6, a novel cable bacteria strain isolated from the Yaquina Bay estuary in Oregon, USA. Strain YB6 exhibits the hallmark capabilities of cable bacteria, such as long-distance electron transport alongside the presence of the unique NiBiD cofactor. However, detailed morphological characterization reveals a distinct phenotype, with ridges up to two to three times wider than those observed in other described cable bacteria species. Additionally, strain YB6 demonstrates abundant extracellular sheath formation.

Using the clonal culture in combination with long- and short-read sequencing, we successfully generated a closed bacterial genome. ANI and 16S rRNA analysis confirm that strain YB6 belongs to a new cable bacteria species. While AAI, POCP, and phylogenomic analyses show that YB6 is more closely related to the *Ca.* Electrothrix genus than to *Ca.* Electronema, YB6 fits within both genera according to established AAI and POCP genus cutoffs. More closely related genomes are needed to fully resolve this taxonomic placement; in the meantime, we cautiously classify strain YB6 as an early-branching member of *Ca.* Electrothrix.

Genome analysis reveals that strain YB6 has a distinct metabolic potential from other members of the *Ca.* Electrothrix genus, including genes involved in electron transfer, phosphate uptake, carbon assimilation, and adaptation to the marine environment. This suggests that its metabolic capabilities represent a mix of features previously considered exclusive to the two cable bacteria genera. Additionally, strain YB6 displays unique genetic features, such as the absence of periplasmic monoheme cytochromes and the nickel export protein RcnA, both of which are present in all other known cable bacteria. Altogether, these characteristics indicate that strain YB6 represents a completely unique cable bacterium, expanding the horizon of cable bacteria diversity. We propose the name *Ca.* Electrothrix yaqonensis YB6 for this new species.

### A note on the etymology of the species name and the history of the Yaqona people

After consulting with the Confederated Tribes of Siletz Indians to access the history of Yaquina Bay and its indigenous people, we propose to name this newly discovered cable bacteria species yaqonensis, to recognize the people of Yaqona. Closely related to Alsea people living to the south, Yaqona people lived for millennia in a series of closely related villages centered upon what today is known as Yaquina Bay. The term Yaqona, the original Yaquina Bay people’s name for themselves, refers to the river and bay that made up much of their homelands. Beginning in the late 18th century, populations of Native people along the Central Oregon Coast decreased rapidly due to disease and then violent attacks by Hudson’s Bay Company employees. In 1855, the ongoing threats of encroachment or even open warfare led the Yaqona people to sign the Coast Treaty, accepting the creation of the Coast (Siletz) Reservation and the forced relocations of thousands of other indigenous peoples from tribes across western Oregon onto their traditional territories. Ten years later, in 1865, political pressure from settlers and business interests to acquire the valuable port and resources of Yaquina Bay led US President Johnson to sidestep Congress and carve the Reservation in two, opening Yaquina Bay and the surrounding 200,000 acres to settlers and forcing the Yaqona people onto the diminishing Reservation. Despite these injustices, Yaqona families managed to adapt and survive. Today, the descendants of the Yaqona people form part of the Confederated Tribes of Siletz Indians headquartered in nearby Siletz, OR (Confederated Tribes of Siletz Indians, personal communication) (88, 89).

Naming a bacterium ecologically important to the environment, such as cable bacteria, after an indigenous tribe recognizes its historical bond with the land and acknowledges its enduring contributions to ecological knowledge and sustainability. This recognition enriches environmental microbiology with cultural diversity and promotes equity in scientific research.

### Emended description of the genus *Candidatus* Electrothrix Trojan et al. 2016

*Candidatus* Electrothrix spp. are multicellular and filamentous, with lengths of up to multiple centimeters and 10–71 characteristic longitudinal ridges and a shared periplasm connected throughout the entire filament; are electron conducting; typically inhabit the surface layer of brackish and marine sediments; have cells that are 0.4–8.0 µm wide and ca. 3 µm long; may contain polyphosphate inclusions; no sulfur inclusions; movement by gliding or twitching motility.

### Description of *Candidatus* Electrothrix yaqonensis YB6 sp. nov.

*Candidatus* Electrothrix yaqonensis (ya.qon.en’sis, from L. adj. yaqonensis, pertaining to the indigenous name “Yaqon” for Yaquina Bay, where the strain was isolated from). This taxon is represented by strain YB6, cultivated from estuarine sediment with a salinity of ~30. Growth is filamentous (up to several centimeters), with individual cells of 0.7–1.2 µm in diameter and 2.6–5.3 µm in length. Filaments contain a small number (between 10 and 13) of pronounced and wide parallel ridge compartments (minimum and maximum average ridge compartment width of all measured cells: 169 and 282 nm, respectively). Filaments are conductive and harbor the unique nickel cofactor (NiBiD) exclusive to cable bacteria. Filaments can be encased in extracellular sheaths, and extensive sheath production is observed during growth. Growth occurs by electrogenic sulfur oxidation, with oxygen as the terminal electron acceptor in marine conditions. Additionally, genomic analysis indicates that growth may also occur via nitrate reduction through dissimilatory nitrate reduction to ammonium. The G+C content is 51.3%. The taxon is distinguishable by morphology and genome content (accession number GCA_047651235.1).

## Data Availability

Amplicon read data of the clonal culture are available under PRJEB83420 in NCBI/ENA/DDBJ. Metagenomic sequencing data for this study have been deposited at the National Center for Biotechnology Information under BioProject ID PRJNA1160179. Genome information has been deposited in GenBank (CP178758.1, GCA_047651235.1).

## SUPPLEMENTAL MATERIAL

Supplemental figures aem.02502-24-s0001.pdf Figure S1 to S9

Supplemental text aem.02502-24-s0002.txt Annotated genome of YB6.

Supplemental material aem.02502-24-s0003.docx Supplementary Legends for Fig. S1 to S9; Tables S5 to S9.

Table S1 aem.02502-24-s0004.xlsx Accession numbers of cable bacteria and Desulfobulbales 16S rRNA gene sequences used for phylogenetic reconstruction.

Table S2 aem.02502-24-s0005.xlsx Accession numbers of cable bacteria and Desulfobulbales 16S rRNA gene sequences used for the identity matrix.

Table S3 aem.02502-24-s0006.xlsx Accession numbers of cable bacteria and Desulfobulbales genomes used for genus delineation.

Table S4 aem.02502-24-s0007.xlsx Ridge measurements of cross-sections.

Table S10 aem.02502-24-s0008.xlsx Comparison of gene presence and absence in key pathways of cable bacteria.

ASM does not own the copyrights to Supplemental Material that may be linked to, or accessed through, an article. The authors have granted ASM a non-exclusive, world-wide license to publish the Supplemental Material files. Please contact the corresponding author directly for reuse.
