# Supplementary material for: A novel cable bacteria species with a distinct morphology and genomic potential
Source: Appl Environ Microbiol. 2025 Apr 22;91(5):e02502-24. doi: 10.1128/aem.02502-24 (PMC12093952; doi:10.1128/aem.02502-24)
Supplement: Supplemental figures — Figure S1 to S9 [file aem.02502-24-s0001.pdf]

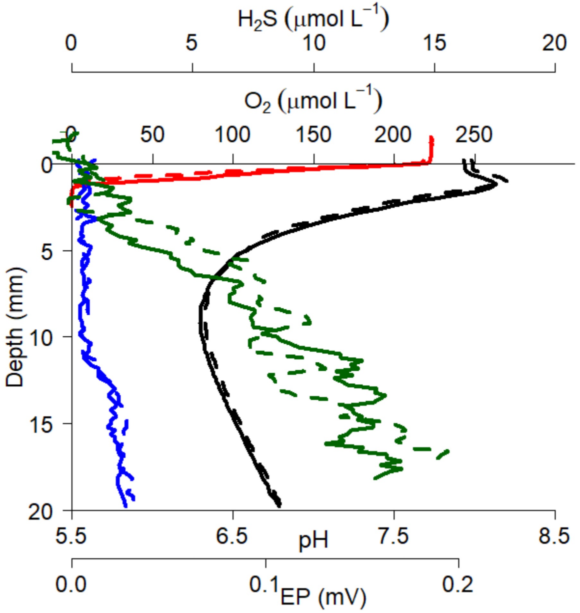

H2S (μmol L−1)
0
5
10
15
20
O2 (μmol L−1)
0
50
100
150
200
250
Depth (mm)
0
5
10
15
20
5.5
6.5
pH
7.5
8.5
0.0
0.1
EP (mV)
0.2

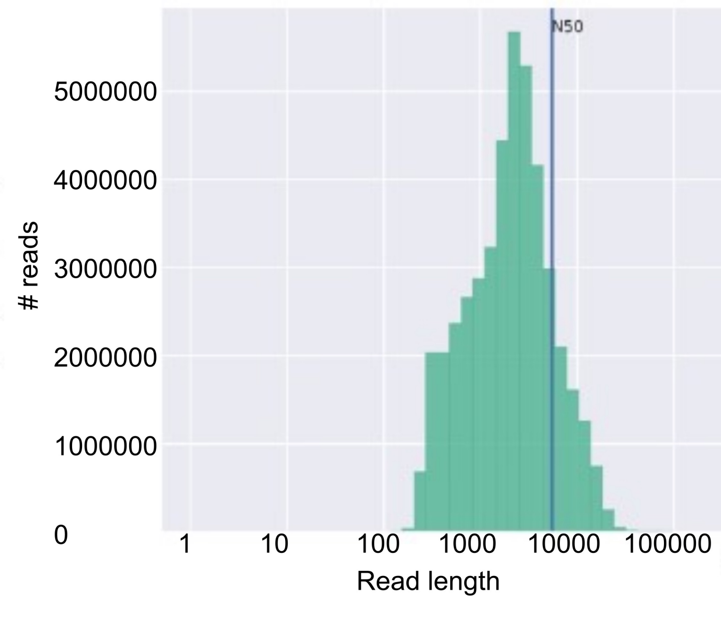

N50
5000000
4000000
3000000
2000000
1000000
0
reads
1
10
100
1000
10000
100000
Read length

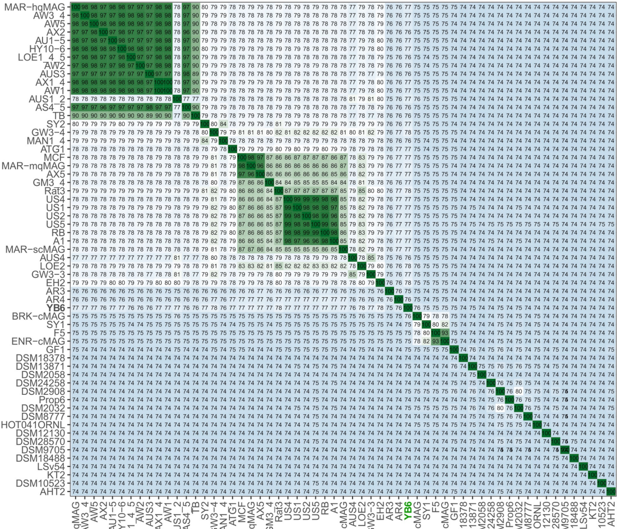
MAR-hqMAG
AW3_4
AW5
AX2
AU1-5
HY10-6
LOE1_4_5
AW2
AUS3
AX1_4
AW1
AUS1_2
AS4_5
TB
SY2
GW3-4
MAN1_4
ATG1
MCF
MAR-mqMAG
AX5
GM3_4
Rat3
US4
US1
US2
US5
RB
A1
MAR-scMAG
AUS4
LOE2
GW3-3
EH2
AR3
AR4
YB6
BRK-cMAG
SY1
F5
ENR-cMAG
GF1
DSM18378
DSM13871
DSM2058
DSM24258
DSM2908
Prop6
DSM2032
DSM8777
HOT041ORNL
DSM12130
DSM28570
DSM9705
DSM18488
LSv54
KT2
DSM10523
AHT2

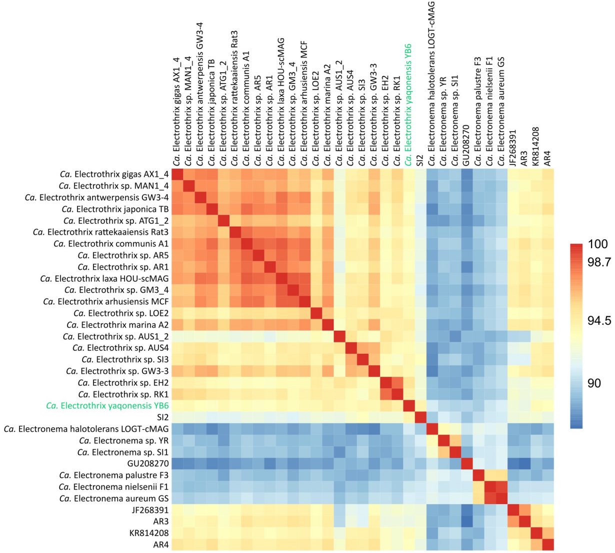
Ca. Electrothrix gigas AX1_4
Ca. Electrothrix sp. MAN1_4
Ca. Electrothrix antwerpensis GW3-4
Ca. Electrothrix japonica TB
Ca. Electrothrix sp. ATG1_2
Ca. Electrothrix rattekaaiensis Rat3
Ca. Electrothrix communis A1
Ca. Electrothrix sp. AR5
Ca. Electrothrix sp. AR1
Ca. Electrothrix laxa HOU-scMAG
Ca. Electrothrix sp. GM3_4
Ca. Electrothrix arhusiensis MCF
Ca. Electrothrix sp. LOE2
Ca. Electrothrix marina A2
Ca. Electrothrix sp. AUS1_2
Ca. Electrothrix sp. AUS4
Ca. Electrothrix sp. SI3
Ca. Electrothrix sp. GW3-3
Ca. Electrothrix sp. EH2
Ca. Electrothrix sp. RK1
Ca. Electrothrix yaqonensis YB6
SI2
Ca. Electronema halotolerans LOGT-cMAG
Ca. Electronema sp. YR
Ca. Electronema sp. SI1
GU208270
Ca. Electronema palustre F3
Ca. Electronema nielsenii F1
Ca. Electronema aureum GS
JF268391
AR3
KR814208
AR4
100
98.7
94.5
90

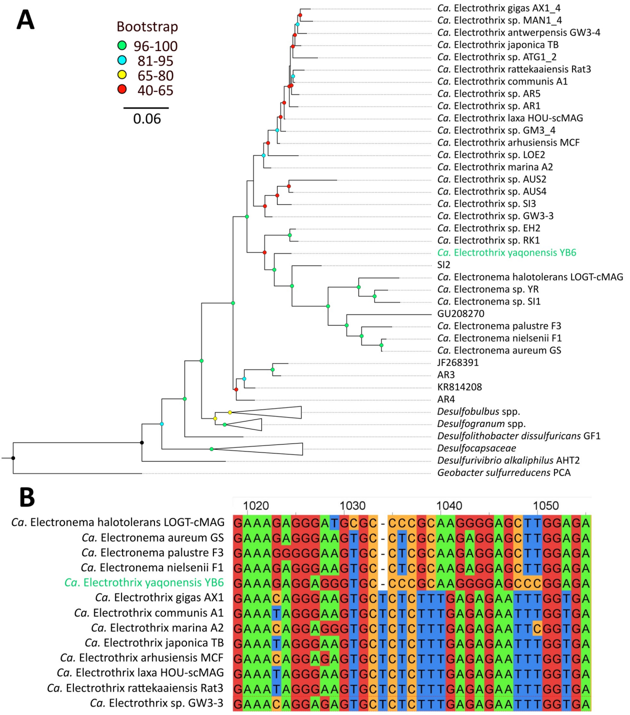
A
Bootstrap
96-100
81-95
65-80
40-65
0.06
Ca. Electrothrix gigas AX1_4
Ca. Electrothrix sp. MAN1_4
Ca. Electrothrix antwerpensis GW3-4
Ca. Electrothrix japonica TB
Ca. Electrothrix sp. ATG1_2
Ca. Electrothrix rattekaaiensis Rat3
Ca. Electrothrix communis A1
Ca. Electrothrix sp. AR5
Ca. Electrothrix sp. AR1
Ca. Electrothrix laxa HOU-scMAG
Ca. Electrothrix sp. GM3_4
Ca. Electrothrix arhusiensis MCF
Ca. Electrothrix sp. LOE2
Ca. Electrothrix marina A2
Ca. Electrothrix sp. AUS2
Ca. Electrothrix sp. AUS4
Ca. Electrothrix sp. SI3
Ca. Electrothrix sp. GW3-3
Ca. Electrothrix sp. EH2
Ca. Electrothrix sp. RK1
Ca. Electrothrix yaqonensis YB6
SI2
Ca. Electronema halotolerans LOGT-cMAG
Ca. Electronema sp. YR
Ca. Electronema sp. SI1
GU208270
Ca. Electronema palustre F3
Ca. Electronema nielsenii F1
Ca. Electronema aureum GS
JF268391
AR3
KR814208
AR4
Desulfobulbus spp.
Desulfogranum spp.
Desulfolithobacter dissulfuricans GF1
Desulfocapsaceae
Desulfurivibrio alkaliphilus AHT2
Geobacter sulfurreducens PCA
B
1020
1030
1040
1050
Ca. Electronema halotolerans LOGT-cMAG GAAAGAGGGATGCGC-CCCGCAAGGGGAGCTTGGAGA
Ca. Electronema aureum GS GAAAGAGGGAAGTGC-CTCGCAAGAGGAGCTTGGAGA
Ca. Electronema palustre F3 GAAAGGGGGAAGTGC-CTCGCAAGAGGAGCTTGGAGA
Ca. Electronema nielsenii F1 GAAAGAGGGAAGTGC-CTCGCAAGAGGAGCTTGGAGA
Ca. Electrothrix yaqonensis YB6 GAAAGAGGAGGGTGC-CCCGCAAGGGGAGCCCGGAGA
Ca. Electrothrix gigas AX1 GAAACAGGGAAGTGCTCTCTTTGAGAGAATTTGGTGA
Ca. Electrothrix communis A1 GAAATAGGGAAGTGCTCTCTTTGAGAGAATTTGGTGA
Ca. Electrothrix marina A2 GAAACAGGAGGGTGCTCTCTTTGAGAGAATTCGGTGA
Ca. Electrothrix japonica TB GAAATAGGGAAGTGCTCTCTTTGAGAGAATTTGGTGA
Ca. Electrothrix arhusiensis MCF GAAACAGGAGAGTGCTCTCTTTGAGAGAATTTGGTGA
Ca. Electrothrix laxa HOU-scMAG GAAATAGGGAAGTGCTCTCTTTGAGAGAATTTGGTGA
Ca. Electrothrix rattekaaiensis Rat3 GAAATAGGGAAGTGCTCTCTTTGAGAGAATTTGGTGA
Ca. Electrothrix sp. GW3-3 GAAACAGGAGAGTGCTCTCTTTGAGAGAATTTGGTGA

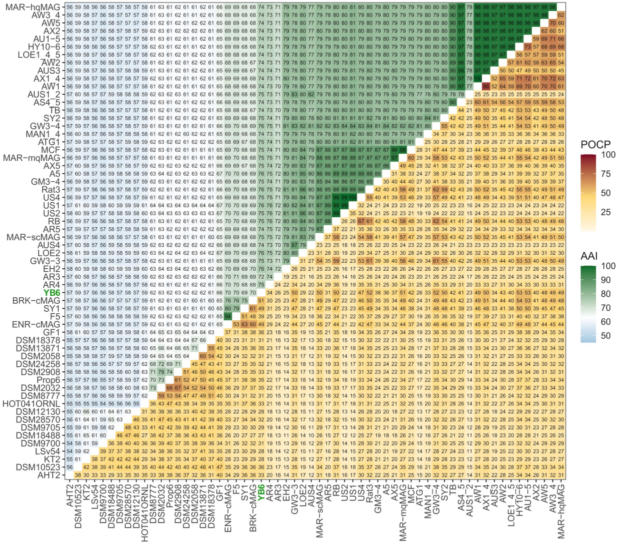
MAR-hqMAG
AW3_4
AW5
AX2
AU1-5
HY10-6
LOE1_4_5
AW2
AUS3
AX1_4
AW1
AUS1_2
AS4_5
TB
SY2
GW3-4
MAN1_4
ATG1
MCF
MAR-mqMAG
AX5
A5
GM3-4
Rat3
US4
US1
US2
RB
AR5
MAR-scMAG
AUS4
LOE2
GW3-3
EH2
AR3
AR4
YB6
BRK-cMAG
SY1
F5
ENR-cMAG
GF1
DSM18378
DSM13871
DSM2058
DSM24258
DSM2908
Prop6
DSM2032
DSM8777
HOT041ORNL
DSM12130
DSM28570
DSM9705
DSM18488
DSM9700
LSv54
KT2
DSM10523
AHT2
POCP
100
75
50
25
AAI
100
90
80
70
60
50

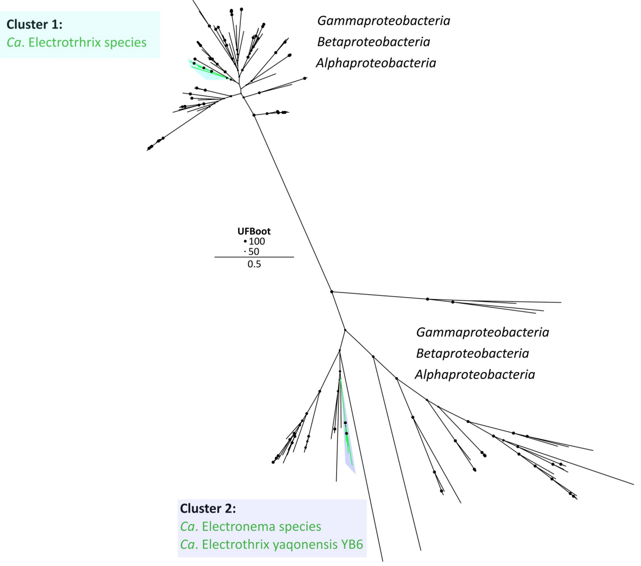

Cluster 1:
Ca. Electrotrhrix species
Gammaproteobacteria
Betaproteobacteria
Alphaproteobacteria
UFBoot
100
50
0.5
Gammaproteobacteria
Betaproteobacteria
Alphaproteobacteria
Cluster 2:
Ca. Electronema species
Ca. Electrothrix yaqonensis YB6

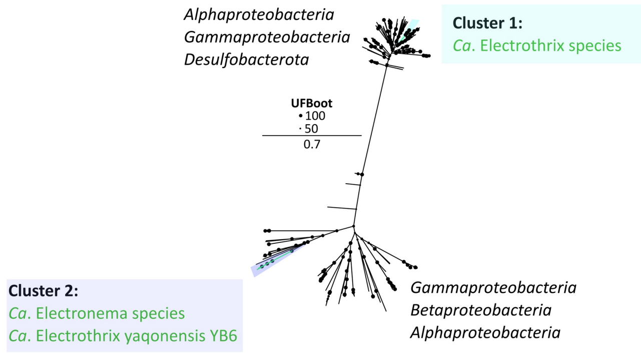
Alphaproteobacteria
Gammaproteobacteria
Desulfobacterota
Cluster 1:
Ca. Electrothrix species
UFBoot
100
50
0.7
Cluster 2:
Ca. Electronema species
Ca. Electrothrix yaqonensis YB6
Gammaproteobacteria
Betaproteobacteria
Alphaproteobacteria

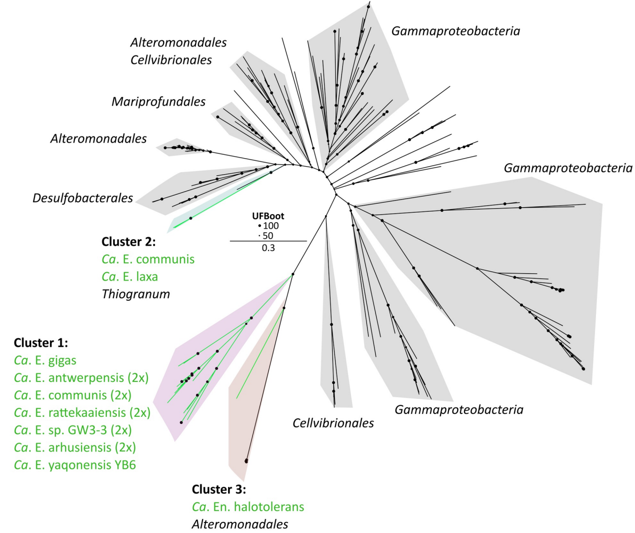
Gammaproteobacteria
Alteromonadales
Cellvibrionales
Mariprofundales
Alteromonadales
Gammaproteobacteria
Desulfobacterales
UFBoot
• 100
· 50
0.3
Cluster 2:
Ca. E. communis
Ca. E. laxa
Thiogranum
Cluster 1:
Ca. E. gigas
Ca. E. antwerpensis (2x)
Ca. E. communis (2x)
Ca. E. rattekaaiensis (2x)
Ca. E. sp. GW3-3 (2x)
Ca. E. arhusiensis (2x)
Ca. E. yaqonensis YB6
Cellvibrionales
Gammaproteobacteria
Cluster 3:
Ca. En. halotolerans
Alteromonadales
