# Supplementary material for: A novel cable bacteria species with a distinct morphology and genomic potential
Source: Appl Environ Microbiol. 2025 Apr 22;91(5):e02502-24. doi: 10.1128/aem.02502-24 (PMC12093952; doi:10.1128/aem.02502-24)
Supplement: Supplemental material — Supplementary Legends for Fig. S1 to S9; Tables S5 to S9. [file aem.02502-24-s0003.docx]

*Supplementary Material*

**A Novel Cable Bacteria Species with a Distinct Morphology and Genomic Potential**

† Anwar Hiralal^1,$^, † Philip Ley^1^ , † Jesper R. van Dijk^1^, † Cheng Li^2,4,5,*^, Dmitrii Pankratov^1^, Jiji Alingapoyil Choyikutty^1^, Galina Pankratova^1^, Jeanine S. Geelhoed^1^, Diana Vasquez-Cardenas^1^, Clare E. Reimers^2^, Filip J.R. Meysman^1,3,*^

^1^ Research group Geobiology, Department of Biology, University of Antwerp, 2610 Wilrijk Antwerp, Belgium

^2^ College of Earth, Ocean and Atmospheric Sciences, Oregon State University, Corvallis, Oregon 97331, USA

^3^ Department of Biotechnology, Delft University of Technology, Van der Maasweg 9, 2629 HZ Delft, The Netherlands

^4^ Integrated Science and Technology, School of Integrated Sciences, James Madison University, MSC 4302, 801 Carrier Drive, Harrisonburg, VA 22807, USA

^5^ Biological & Ecological Engineering, College of Agricultural Science, Oregon State University, Corvallis, OR 97331, USA

^$^ Present address: Department of Functional and Evolutionary Ecology, University of Vienna, Djerassiplatz 1, 1030 Vienna, Austria

† These authors contributed equally

* Corresponding authors:

Cheng Li: Cheng.Li@oregonstate.edu

Filip J. R. Meysman: filip.meysman@uantwerpen.be

## **Supplementary figures**


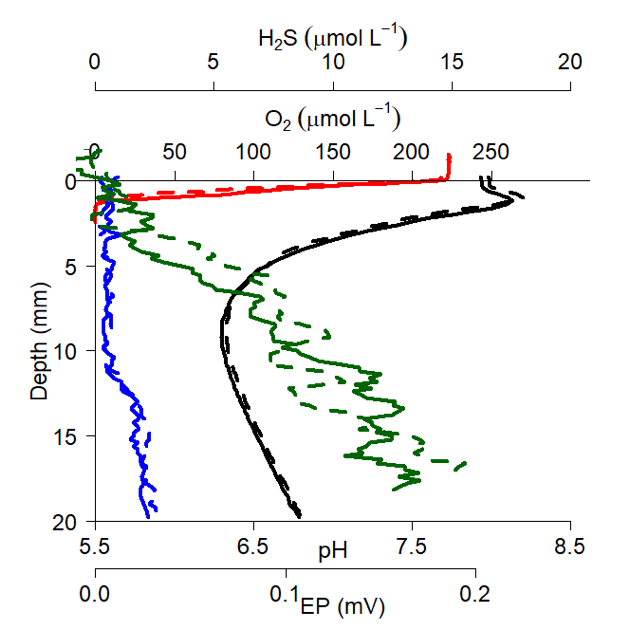


**Figure S1:** Microsensor depth profiles of core containing cable bacteria YB6. Oxygen is represented in red, electric potential in green, pH in black, and ∑H_2_S in blue. Depth profile shows a distinct cable bacteria fingerprint as a result of e-SOx, with a pH maximum at the sediment water interface, a pH minimum and a suboxic zone, where no oxygen and sulfide are detectable in the porewater. Additionally, an electrical potential difference is visible across the suboxic zone. Duplicate profiles are shown in dashed lines.


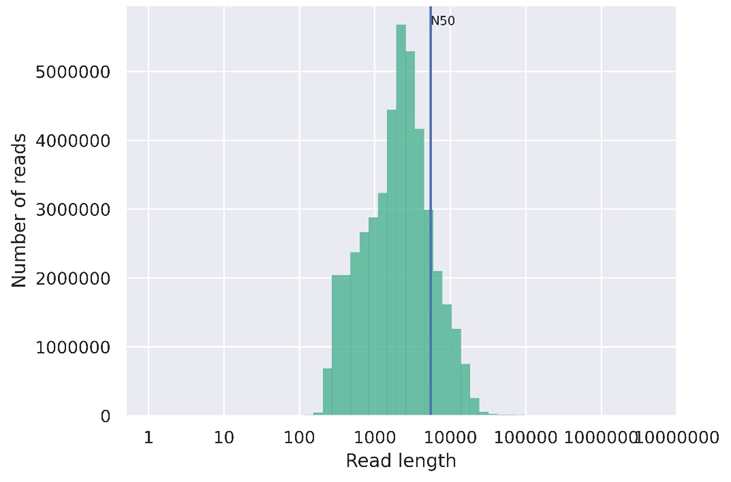


**Figure S2.** Log-scaled read length histogram of the YB6 sediment sample from Nanopore sequencing. N_50_ = 5465 bp. Figure obtained using NanoPlot (De Coster et al., 2018)

**Figure S3.** Percentage of Average Nucleotide Identity (ANI) in cable bacteria and reference genomes. Only strain names are indicated (see Table S1). Strain YB6 is indicated in green.

**Figure S4.** Distance matrix comparing 16S rRNA gene sequence identities of cable bacteria. Colors indicate % sequence identity.

**Figure S5.** A) 16S rRNA phylogeny and multiple alignment A**)** Maximum-likelihood 16S rRNA gene tree of *Ca*. Electrothrix yaqonensis YB6 and related reference cable bacteria and close relatives. The tree was run in IQtree with 1000 ultrafast bootstraps using bestfit model GTR+F+I+G4. B) 16S region showing closer alignment of YB6 with *Ca*. Electronema compared to *Ca*. Electrothrix species. Sequences were aligned with MUSCLE (Edgar, 2004).

**Figure S6**. Percentage of Average Amino Acid Identity (AAI) and Percentage of Conserved Proteins (POCP) in cable bacteria and reference genomes. Only strain names are indicated (see Table S1). Strain YB6 is indicated in green.

**Figure S7**: Maximum likelihood phylogeny of CytB561 sequences of cable bacteria and RefSeq database hits. Made using IQtree using the best-fit model (LG+F+I+G4). Cable bacteria sequences are indicated in green branches. The sequence of strain YB6 clusters with sequences of *Ca*. Electronema (cluster 2). Note that all other branches belong to the *Gammaproteobacteria*, *Betaproteobacteria* or *Alphaproteobacteria*.

**Figure S8**: Maximum likelihood phylogeny of PstA sequences of cable bacteria and RefSeq database hits. Made using IQtree using the best-fit model (LG+I+G4). Cable bacteria sequences are indicated in green branches. The sequence of strain YB6 clusters with sequences of *Ca*. Electronema (cluster 2). Other than cable bacteria, the bottom side of the tree contains sequences of *Gammaproteobacteria*, *Alphaproteobacteria* and *Betaproteobacteria*, while the top part contains sequences of *Alphaproteobacteria*, *Gammaproteobacteria* and *Desulfobacterota*.

**Figure S9**: Maximum likelihood phylogeny of NhaA sequences of cable bacteria and RefSeq database hits. Made using IQtree using the best-fit model (LG+F+I+G4). Cable bacteria sequences are indicated in green branches. Different clusters are indicated.

## **Supplementary Tables**

**Table S1.** Accession numbers of cable bacteria and Desulfobulbales 16S rRNA gene sequences used for phylogenetic reconstruction.

*Table S1 is available as a separate Excel file*

**Table S2.** Accession numbers of cable bacteria and Desulfobulbales 16S rRNA gene sequences used for the identity matrix.

*Table S2 is available as a separate Excel file*

**Table S3.** Accession numbers of cable bacteria and Desulfobulbales genomes used for genus delineation.

*Table S3 is available as a separate Excel file*

**Table S4.** Ridge measurements of cross-sections

*Table S4 is available as a separate Excel file*

**Table S5**. Michaelis–Menten kinetic parameters obtained for the oxygen-reducing activity of YB6.

| Sample | *K’_m_*, µM | *I_max_*, nA |  |
| --- | --- | --- | --- |
|  |  |  |  |
| 1 | 4.9 ± 0.4 | 152.0 ± 3.7 |  |
| 2 | 2.9 ± 0.3 | 56.1 ± 1.6 |  |
| 3 | 5.8 ± 1.1 | 4.8 ± 0.3 |  |

**Table S6.** Annotation of the principal vibrational modes in cable bacteria YB6. References for annotation.

| Wavenumber | Assignment | Ref |
| --- | --- | --- |
| 373 cm^-1^ | Nickel-sulfur ligated NiBiD cofactor: ν(Ni-S)_sym_ − Ni-S stretching | 1, 2 |
| 488 cm^-1^ | Nickel-sulfur ligated NiBiD cofactor: Ni-cofactor: ν(C-S) + ring deformation | 1, 2 |
| 749 cm^-1^ | Cytochromes: ν_15_ − Pyrrole breathing | 3, 4 |
| 1130 cm^-1^ | Cytochromes: ν_22_ − vibrations of side radicals C_b_-CH_3_ | 5, 6 |
| 1170 cm^-1^ | Cytochromes: ν_30_ – asymmetric stretching pyrrole half-ring | 5, 6 |
| 1230 cm^-1^ | Cytochrome *c*: ν_14_ – C_m_-H in-plane bending | 5, 6 |
| 1315 cm^-1^ | Cytochrome *c*: ν_21_ – δ(C_m_H) | 5, 6 |
| 1360 cm^-1^ | Cytochromes: ν_4_ − symmetric pyrrole half-ring stretching | 3, 4 |
| 1399 cm^-1^ | Cytochromes: ν_20_ – stretching of pyrrole quarter-ring | 5, 6 |
| 1588 cm^-1^ | Cytochromes: ν_19_ − ν(C_α_C_m_)_asym_ | 3, 4 |
| 1640 cm^-1^ | Cytochromes: ν_10_ − ν(C_α_C_m_)_asym_ | 3, 4 |

**Table S7.** Nanopore sequencing output overview. Reads were filtered for an average Q20 and a size >10 kb.

|  | YB6 |
| --- | --- |
| Nanopore flow cell | FLO-PRO114M |
| Sequencing kit | SQK-LSK114 |
| Raw bases (bp) | 149,375,104,373 |
| Raw reads | 44,677,451 |
| Bases after QC (bp) | 34,377,926,343 |
| Reads after QC | 4,334,464 |
| Average read length (bp) | 3,343 |
| Read length N50 (bp) | 5,465 |

**Table S8.** Illumina sequencing overview.

|  | YB6 |
| --- | --- |
| Sequencing platform | Illumina NovaSeq |
| Paired raw bases | 7,694,555,018 |
| Paired raw reads | 50,957,318 |
| Paired bases after QC (bp) | 6,552,234,884 |
| Paired reads after QC | 44,542,158 |

**Table S9:** Genome features of *Ca*. Electrothrix yaqonensis YB6

|  | *Ca*. Electrothrix yaqonensis YB6 |
| --- | --- |
| Genome size | 3,738,687 |
| Genome structure | circular |
| Coding sequences | 3,402 |
| Protein coding genes | 3,323 |
| 16S‑23S‑5S rRNA loci | 2 |
| GC content (%) | 51.3 |

**Table S10.** Comparison of gene presence and absence in key pathways of cable bacteria.

*Table S10 is available as a separate Excel file*

**Table S11.** Protologue table for *Ca.* Electrothrix yaqonensis.

| **Species name** | *Ca.* Electrothrix yaqonensis |
| --- | --- |
| **Genus name** | *Ca.* Electrothrix |
| **Specific epithet** | yaqonensis |
| **Type strain** | YB6 |
| **Type species of the genus** | *Ca.* Electrothrix yaqonensis |
| **Species etymology** | *Ca.* Electrothrix yaqonensis sp. nov.: ya.qo.nen’sis, from L. adj. yaqonensis, pertaining to Yaquina Bay (USA), referred to by the native Yaqona tribe as "Yaqon," where the organism was isolated from. |
| **Species status** | sp. nov. |
| **Type strain** | YB6 |
| **Assembly project** | PRJNA1160179 |
| **Genome accession number** | CP178758 |
| **Genome topology** | Circular |
| **Genome Size (bp)** | 3,738,687 |
| **G+C%** | 51.31 |
| **Country of origin** | The United States of America |
| **Region of origin** | Oregon |
| **Sample source** | Sediment |
| **Geographical location** | Yaquina Bay |
| **Latitude** | 44.618344°N |
| **Longitude** | -124.041247°W |
| **Sample Depth** | Intertidal |
| **Assembly method** | Hybrid |
| **Sequencing technology** | Oxford Nanopore R10.4.1 and Illumina NovaSeq |
| **Binning** | Not performed |
| **Assembly software used** | Flye 2.9 |
| **Habitat** | Intertidal zone (coastal habitat) |
| **Miscellaneous, extraordinary features relevant for the description** | Circular genome obtained from a metagenomic sample of clonal cable bacterium enrichment YB6 |

**References**

1. Johnson, M. K. (2004). “Vibrational spectra of dithiolene complexes,” in Dithiolene Chemistry: Synthesis, Properties, and Applications, ed. E. Stiefel (Hoboken, NJ, United States of America: John Wiley & Sons, Inc), 213–266. doi: 10.1002/0471471933. Chapter 4.
2. Smets, B., Boschker, H.T.S., Wetherington, M.T., Lelong, G., Hidalgo-Martinez, S., Polerecky, L., Nuyts, G., De Wael, K. and Meysman, F.J.R. (2024). Multi-wavelength Raman microscopy of nickel-based electron transport in cable bacteria. *Front. Microbiol.* 15:1208033. doi: 10.3389/fmicb.2024.1208033.
3. Virdis, B., Millo, D., Donose, B. C., and Batstone, D. J. (2014). Real-time measurements of the redox states of c-type cytochromes in electroactive biofilms: a confocal resonance Raman microscopy study. PLoS One 9:89918. doi: 10.1371/journal.pone.0089918
4. Milazzo, L., Tognaccini, L., Howes, B. D., and Smulevich, G. (2018). Probing the nonnative states of cytochrome c with resonance Raman spectroscopy: a tool for investigating the structure–function relationship. J. Raman Spectrosc. 49, 1041–1055. doi: 10.1002/jrs.5315
5. Berezhna, S., Wohlrab, H., & Champion, P. M. (2003). Resonance Raman investigations of cytochrome c conformational change upon interaction with the membranes of intact and CA2+-exposed mitochondria. *Biochemistry*, *42*(20), 6149–6158. doi.org/10.1021/bi027387y
6. Hu, S., Spiro, T. G., Morris, I. K., Singh, J. P., & Smith, K. M. (1993). Complete Assignment of Cytochrome c Resonance Raman Spectra via Enzymatic Reconstitution with Isotopically Labeled Hemes. *Journal of the American Chemical Society*, *115*(26), 12446–12458. doi.org/10.1021/ja00079a028
